# A spatial model of autophosphorylation of CaMKII predicts that the lifetime of phospho-CaMKII after induction of synaptic plasticity is greatly prolonged by CaM-trapping

**DOI:** 10.3389/fnsyn.2025.1547948

**Published:** 2025-04-04

**Authors:** Thomas M. Bartol, Mariam Ordyan, Terrence J. Sejnowski, Padmini Rangamani, Mary B. Kennedy

**Affiliations:** ^1^The Salk Institute for Biological Studies, La Jolla, CA, United States; ^2^Department of Neurobiology, University of California, San Diego, La Jolla, CA, United States; ^3^Department of Mechanical and Aerospace Engineering, University of California, San Diego, La Jolla, CA, United States; ^4^Division of Biology and Biological Engineering, California Institute of Technology, Pasadena, CA, United States

**Keywords:** stochastic simulation, synaptic plasticity, autophosphorylation, CaMKII, LTP induction

## Abstract

Long-term potentiation (LTP) is a biochemical process that underlies learning in excitatory glutamatergic synapses in the Central Nervous System (CNS). A critical early driver of LTP is autophosphorylation of the abundant postsynaptic enzyme, Ca^2+^/calmodulin-dependent protein kinase II (CaMKII). Autophosphorylation is initiated by Ca^2+^ flowing through NMDA receptors activated by strong synaptic activity. Its lifetime is ultimately determined by the balance of the rates of autophosphorylation and of dephosphorylation by protein phosphatase 1 (PP1). Here we have modeled the autophosphorylation and dephosphorylation of CaMKII during synaptic activity in a spine synapse using MCell4, an open source computer program for creating particle-based stochastic, and spatially realistic models of cellular microchemistry. The model integrates four earlier detailed models of separate aspects of regulation of spine Ca^2+^ and CaMKII activity, each of which incorporate experimentally measured biochemical parameters and have been validated against experimental data. We validate the composite model by showing that it accurately predicts previous experimental measurements of effects of NMDA receptor activation, including high sensitivity of induction of LTP to phosphatase activity *in vivo,* and persistence of autophosphorylation for a period of minutes after the end of synaptic stimulation. We then use the model to probe aspects of the mechanism of regulation of autophosphorylation of CaMKII that are difficult to measure *in vivo*. We examine the effects of “CaM-trapping,” a process in which the affinity for Ca^2+^/CaM increases several hundred-fold after autophosphorylation. We find that CaM-trapping does not increase the proportion of autophosphorylated subunits in holoenzymes after a complex stimulus, as previously hypothesized. Instead, CaM-trapping may dramatically prolong the lifetime of autophosphorylated CaMKII through steric hindrance of dephosphorylation by protein phosphatase 1. The results provide motivation for experimental measurement of the extent of suppression of dephosphorylation of CaMKII by bound Ca^2+^/CaM. The composite MCell4 model of biochemical effects of complex stimuli in synaptic spines is a powerful new tool for realistic, detailed dissection of mechanisms of synaptic plasticity.

## Introduction

**Figure 1:**
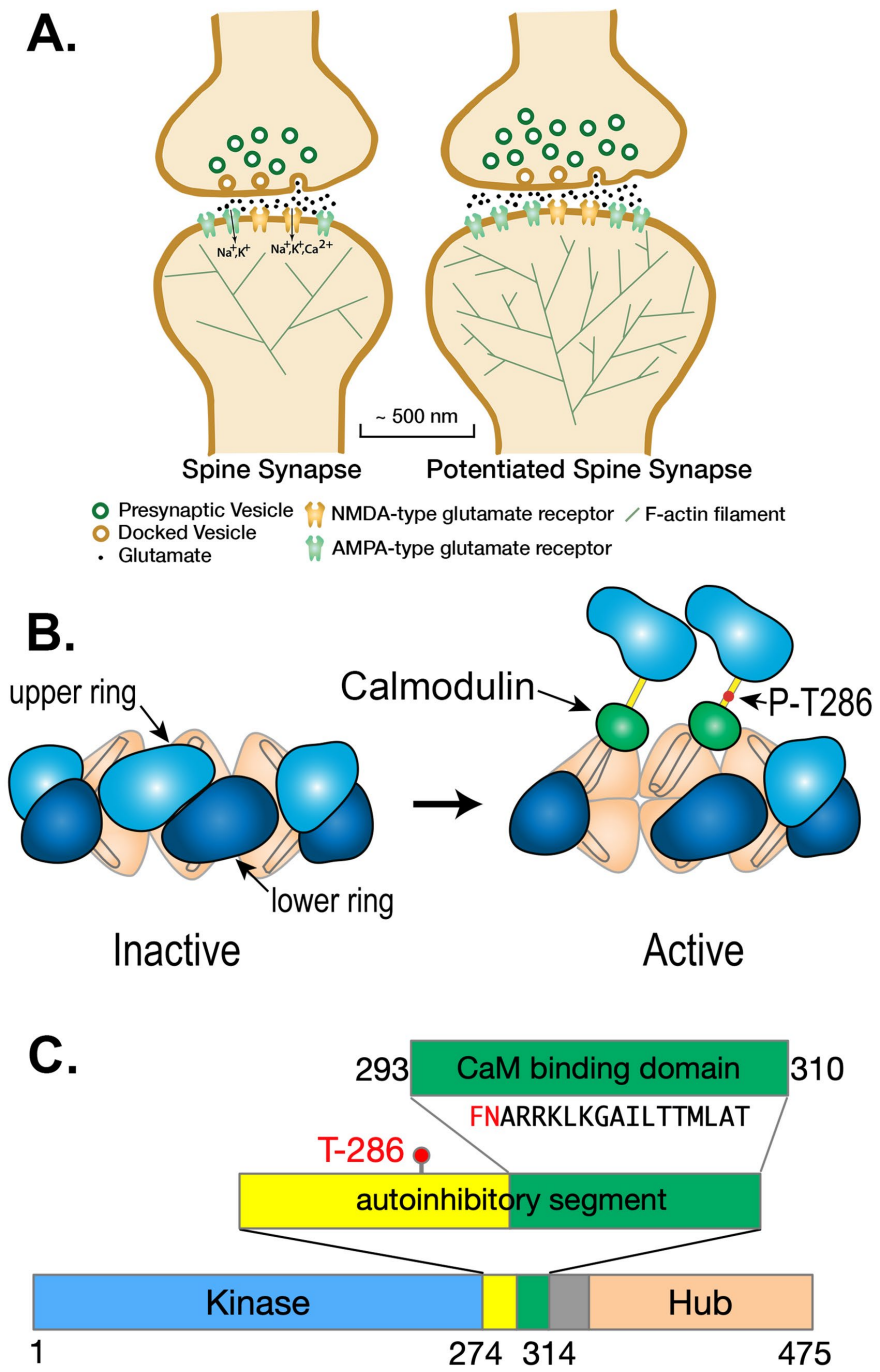
**(A)** Schematic diagram of a spine synapse. AMPA-type glutamate receptors (AMPARs, teal) produce a depolarizing Na^+^/K^+^ current upon binding glutamate released from presynaptic vesicles after arrival of an axonal action potential (AP). NMDA-type glutamate receptors (NMDARs, gold) contain a channel that is opened by the concurrence of glutamate release and strong depolarization (e.g., a back-propagating action potential [bAP]). Potentiated synapses (right) contain more AMPARs resulting in a larger excitatory postsynaptic potential (EPSP) upon release of glutamate. The spines of potentiated synapses increase in size and contain a more highly branched actin cytoskeleton. **(B)** Ca^2+^/calmodulin-dependent protein kinase II (CaMKII) is a predominantly dodecameric holoenzyme in which 12 subunits are bound together by the interactions among the hub domains of each subunit. When Ca^2+^/calmodulin (Ca^2+^/CaM) binds to two adjacent subunits in one of the hexameric rings, a threonine residue (Thr286) within one of the two subunits is autophosphorylated by the other subunit acting as a kinase. Autophosphorylation is believed to occur in one direction around the hexameric ring. Autophosphorylation of Thr 286 causes the subunit to remain active even when the Ca^2+^ concentration falls and calmodulin (CaM) unbinds. (Modified from Figure 7 of Rosenberg et al., 2005). **(C)** Domain diagram of a subunit of CaMKII illustrating the catalytic domain (Kinase), the autoinhibitory segment with the CaM-binding domain, and the Hub domain. When the kinase is inactive, the inhibitory segment lies within the site that binds ATP and protein substrates. Binding of Ca^2+^/CaM to the CaM-binding domain moves the inhibitory segment out of the substrate-binding pocket allowing ATP to bind to the subunit. Thr-286, which can be autophosphorylated, as described in **(B)**, is contained within the autoinhibitory segment. Autophos phorylation unmasks a phenylalanine (F) and a glutamine (N) in the CaM-binding domain (shown in red), enabling a large increase in the affinity of Ca^2+^/CaM, known as CaM-trapping.

Memories are stored in the brain through creation of new neural networks that are formed by strengthening excitatory glutamatergic synapses between neurons that are activated together during an experience (Magee and Johnston, 1997; Markram et al., 1997; Bi and Poo, 1998). Each excitatory pyramidal neuron in the forebrain contains approximately 10,000 excitatory glutamatergic synapses arrayed along several dendrites that reach into the surrounding brain tissue. Most excitatory synapses are comprised of a release site from a presynaptic axon that makes a specialized synaptic contact with a postsynaptic spine, which is a small tubular membrane extension with a bulbous head (Figure 1A). The spine contains highly integrated biochemical machinery that regulates synaptic strength by controlling the size of the spine head and the number of α-amino-3-hydroxy-5-methyl-4-isoxazolepropionic acid (AMPA)-type glutamate receptors (AMPARs) located at the synaptic site (Kennedy et al., 2005; Opazo and Choquet, 2011; Kennedy, 2013; Biederer et al., 2017). Activation of N-methyl-D-aspartate-type glutamate receptors (NMDARs) at synapses in the CNS triggers changes in synaptic strength that underlie memory formation in response to strong synaptic stimuli (Bliss and Collingridge, 1993; Nicoll and Schulman, 2023). Precise kinetic control of this machinery determines the amplitude of changes in synaptic strength that are the basis of new neural networks.

### Spine synapses and LTP

Spines are, on average, ~1–2 μm in length. The diameters of spine heads are highly variable, as are their volumes (Harris and Weinberg, 2012; Bartol et al., 2015a; see Bartol et al., 2015b). The median volume of spines in stratum radiatum of hippocampal area CA1 (the source of our model spine) is ~0.017 μm^3^ or ~ 17 × 10^−18^ liters (Bartol et al., 2015a). The synaptic membrane contacts between axon release sites and spines are roughly circular and their median diameter is ~200 nm (Bartol et al., 2015a). The presynaptic side of the contact contains sites at which vesicles dock at the membrane and release glutamate probabilistically when the axon fires an action potential. The postsynaptic contact is undergirded by the postsynaptic density (PSD), which immobilizes the synaptic glutamate receptors and anchors a dense network of cytosolic regulatory proteins attached to the membrane (Biederer et al., 2017; Harris and Weinberg, 2012; Kennedy, 2000). The strength of a synapse is highly correlated with the volume of the spine head, as well as the area of the PSD and presynaptic active zone (Bourne and Harris, 2007). Larger spine heads contain more AMPA-type glutamate receptors and a larger PSD. Thus, they exhibit a larger excitatory postsynaptic potential when glutamate is released from the presynapse (Bartol et al., 2015a). The regulatory biochemical machinery in spines is capable of responding to the frequency of synaptic activation by strengthening (enlarging) or weakening (shrinking) the synapse (Kennedy, 2013; Malenka and Bear, 2004). The biochemical mechanisms that allow the frequency of presynaptic activity to be translated precisely into synaptic strengthening or weakening that faithfully encodes memories are still incompletely understood. The reactions that lead to functional and structural changes in spines are triggered by Ca^2+^ entering the spine through the ion channels of NMDA-type glutamate receptors (NMDARs) (Figure 1A). Physiological studies show that these functional and structural changes are exquisitely tightly regulated by the frequency and amplitude of Ca^2+^ entry (Sjostrom and Nelson, 2002; Jedrzejewska-Szmek et al., 2023).

A major target of Ca^2+^ entering the spines of cortical and hippocampal excitatory synapses is Ca^2+^/calmodulin-dependent protein kinase II (CaMKII), which, as its name suggests, is activated when it binds Ca^2+^-bound calmodulin (Ca^2+^/CaM) formed when the concentration of Ca^2+^ rises above its baseline value (~100 nM, Pepke et al., 2010) (Figure 1B). Activation of CaMKII has been shown to play a crucial role in long term potentiation (LTP) of excitatory synaptic strength in the hippocampus, and therefore in spatial learning and memory (Silva et al., 1992; Giese et al., 1998). The regulation of CaMKII by Ca^2+^/CaM has also been studied extensively *in vitro* (Pepke et al., 2010; Miller and Kennedy, 1986; Meyer et al., 1992; Hell, 2014; Bayer and Schulman, 2019). Activated CaMKII subunits within the holoenzyme become autophosphorylated at Thr 286 within the first several seconds of Ca^2+^ influx, eventually enabling binding of these subunits to the GluN2B subunits of NMDARs (Tullis et al., 2023; Chen et al., 2024). Activation of CaMKII by autophosphorylation also enables phosphorylation of several additional synaptic proteins (Oh et al., 2004; Araki et al., 2015; Walkup et al., 2015; Diering and Huganir, 2018). These events set in motion a cascade of reactions that lead to changes in synaptic strength, the magnitude of which depend on the frequency of presynaptic action potentials (APs) and the presence of other regulatory modulators that can influence the sensitivity of the synapse to plastic changes (Kennedy et al., 2005; Kennedy, 2013; Blitzer et al., 2005).

Modeling of the dynamics of synaptic mechanisms, including activation of CaMKII, has been carried out at different levels of abstraction and mechanistic realism to address different questions. Previous models have often employed simplifications of complex spatial mechanisms, and/or included coarse-grained variables to represent a series of linked mechanisms over several time scales. Many of these have been deterministic compartmental models that rely on differential equations assuming well-mixed conditions in linked small compartments (Holmes, 2000; Bhalla, 2002; Hayer and Bhalla, 2005; Singh and Bhalla, 2018). In one previous modeling study designed to test the limits of the popular hypothesis that CaMKII constitutes a “bistable” switch that would outlast molecular turnover (Miller et al., 2005), the authors searched for parameters that would produce such a stimulus-dependent “bistable” switch composed of CaMKII and the protein phosphatase PP1 (e.g., Lisman, 1985). However, the parameter ranges they identified as necessary for this form of irreversible “bistability” are not consistent with data regarding the concentrations of these proteins in synaptic spines. Our study also does not predict the existence of a bistable switch composed of CaMKII and PP1 that would outlast molecular turnover.

In another study, Chang et al. (2019), using fluorescently-labeled CaM and CaMKII and fluorescence resonance energy transfer (FRET), measured CaM binding to CaMKII in a spine within the first several seconds of a “high frequency” stimulus produced by repeated glutamate uncaging in the absence of Mg^2+^. The uncaging would produce a considerably stronger Ca^2+^ stimulus than that resulting from our two epoch stimulus. They reported that the first stimulus saturated CaM binding to wild type CaMKII, with no further increase upon repeated stimuli. Despite the different stimulus and the small perturbations introduced by the use of FRET, the coarse-grained model suggested by these authors to explain their results is compatible with our more detailed model (see Discussion). The group of Yasuda, also used fluorescently-labeled derivatives of calmodulin and CaMKII to experimentally probe activation of CaMKII by Ca^2+^ influx into spines induced by local uncaging of light-sensitive glutamate (Chang et al., 2019; Chang et al., 2017). Their measured time courses of activation and decay of binding of CaM to CaMKII and of phosphorylation and activation of CaMKII are similar, although not identical, to our findings.

Here, we have used modeling to study biochemical responses in spine synapses that are dictated by actual biological structures. Although deterministic methods are useful and appropriate for modeling relatively large compartments that are well-mixed, the biochemical reactions that initiate synaptic plasticity *in vivo* occur within extremely small spines which contain small numbers (ten to a few hundred) of spatially-organized signaling proteins (see Kennedy, 2000; Zador et al., 1990). Thus, the biochemical molecular mechanisms initiated in spines in the first few minutes of intense synaptic activity involve binding and chemical reactions that occur in a discontinuous, stochastic fashion that is not well represented by differential equations. They also occur in spatially organized compartments that do not satisfy the “well-mixed” assumptions required for the use of deterministic methods to model reaction dynamics. The ability of MCell4 to efficiently represent both the spatial organization of molecular interactions in small spaces and the stochastic nature of state changes (Husar et al., 2024) makes it an especially useful modeling tool for probing the details of synaptic molecular mechanisms that are driven *in vivo* by the fluctuating, probabilistic Ca^2+^ signals that arise in spines during the first few minutes of synaptic activity. For example, the agent-based, “network free” methods employed in MCell4 with BioNetGen Language (BNGL) allow us to avoid simplifying assumptions regarding the interactions of Ca^2+^, CaM, and CaMKII (~18^12^ states) described below. Methods based on differential equations or population-based stochastic methods [e.g., any method based on the Gillespie Stochastic Simulation Algorithm (SSA), (Gillespie, 1977; Gillespie, 2007)] would not be able to capture the combinatorial complexity inherent in these interactions without simplifying assumptions.

We constructed a composite model of activation of CaMKII in a spine by combining four previously published models. We first show that the composite model produces the observed behavior of NMDARs and resulting Ca^2+^ influx into a spine. We validate it as a model of synaptic activation of CaMKII by showing that it predicts *a priori* aspects of phosphorylation and dephosphorylation of CaMKII *in vivo* that have been experimentally measured, specifically high sensitivity to protein phosphatase activity (e.g., Brown et al., 2000) and persistence of autophosphorylation for a few minutes after the end of the synaptic stimulus (Lee et al., 2009). We then use the model to test hypotheses about the role of CaM-trapping (described below) in regulation of synaptic plasticity that are not easily testable experimentally.

### Medical relevance

Modeling of the precise kinetics of synaptic regulatory mechanisms will have medical relevance. Several intractable mental and neurological illnesses, including Alzheimer’s disease (Selkoe, 2011), involve disruptions in mechanisms of synaptic regulation (Mullins et al., 2016; Scolnick, 2017; Rammes, 2023) that can produce relatively subtle, but devastating, changes in the timing of synaptic reactions. Given the central role of CaMKII activation for synaptic plasticity, it is important to understand the detailed biochemical dynamics of the Ca^2+^-CaM-CaMKII signaling axis within spines to inform the design of new therapeutics to treat these illnesses.

### Structure of CaMKII

CaMKII is a predominantly dodecameric holoenzyme comprised of 12 individual catalytic subunits that are organized via their “hub” domains into two hexameric rings stacked together (Figures 1B,C; Bennett et al., 1983; Rosenberg et al., 2005; Chao et al., 2011). The kinase activity of each subunit is activated independently by binding of Ca^2+^/CaM to its CaM-binding domain, which moves the inhibitory domain away from the catalytic pocket and thus activates kinase activity (Colbran et al., 1989). In addition, the subunits can undergo autophosphorylation in which an active subunit phosphorylates a specific threonine residue (Thr 286) within its neighboring subunit if that subunit also has Ca^2+^/CaM bound to it (Miller and Kennedy, 1986; Miller et al., 1988; Schworer et al., 1988; Thiel et al., 1988; Hanson et al., 1994). Phosphorylation of Thr 286 blocks inhibition by the regulatory domain (Colbran et al., 1989); therefore, each autophosphorylated subunit stays active as long as Thr 286 remains phosphorylated, and can, in turn, phosphorylate and activate its neighboring subunit when the neighbor binds Ca^2+^/CaM. Thr 286 can be dephosphorylated by either of two broad-specificity protein phosphatases, phosphatase 1 (PP1) or -2a (PP2a); however, PP1 activity predominates in spines (Shields et al., 1985; Strack et al., 1997; Watanabe et al., 2001; Monroe and Heathcote, 2013; Platholi and Hemmings, 2021; Sandal et al., 2021). The subunits are encoded by four closely related genes that express subunit variants α, β, γ, and 𝛿 (Gaertner et al., 2004). In the forebrain, α and β-subunits predominate, with heterogeneous holoenzymes having an ~3:1 ratio of α to β (Bennett et al., 1983). Because α-subunits are synthesized and assembled into holoenzymes in the dendrites as well as in the cell soma, synaptic holoenzymes have a high level of α-subunits (Burgin et al., 1990). In this study, we have modeled activation of dodecameric holoenzymes comprised solely of α-subunits.

**Figure 2:**
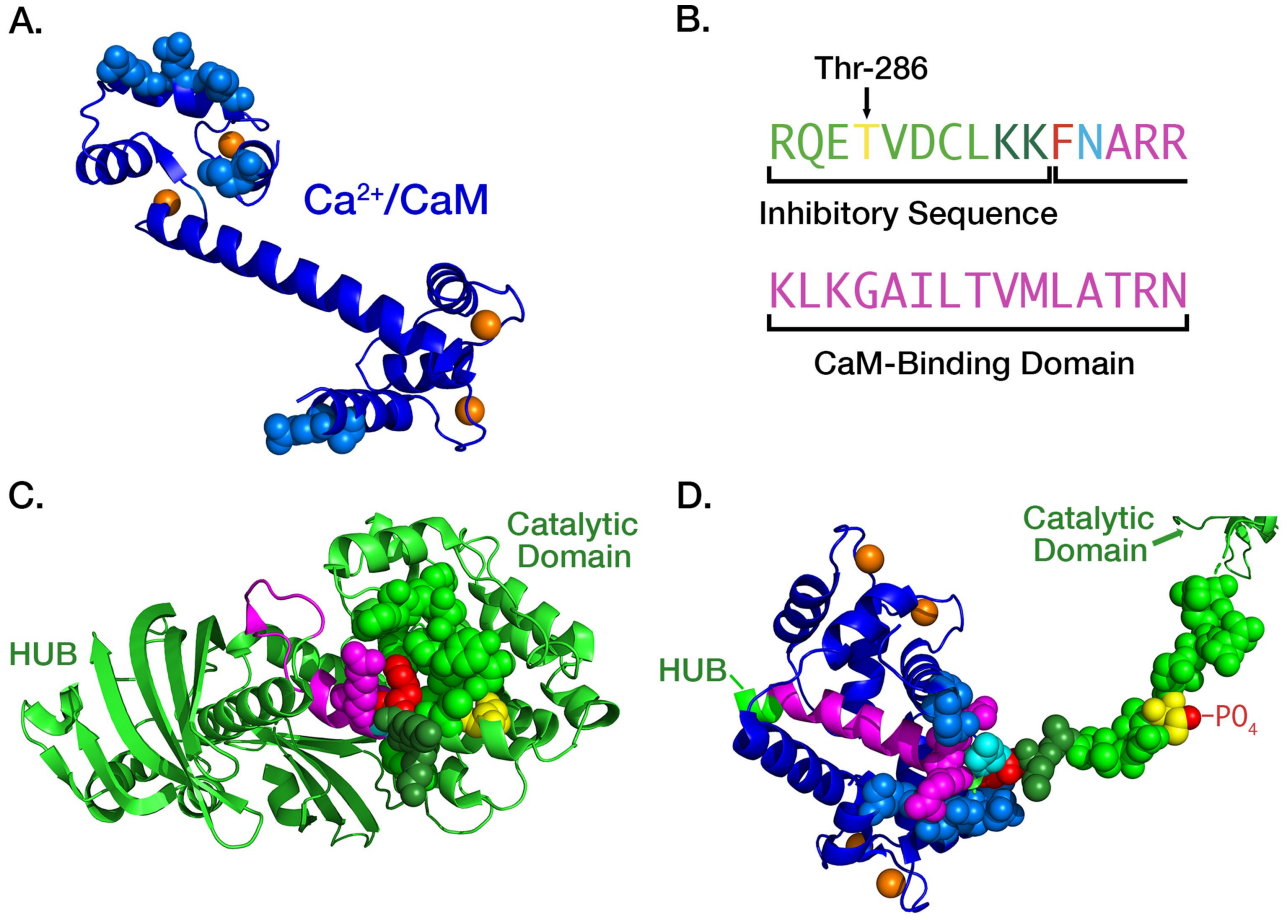
**(A)** Ribbon diagram of the atomic structure of CaM with four bound Ca^2+^ ions (orange), two each at its N- and C- termini. The atoms of residues that are bound tightly to the inhibitory segment of CaMKII during trapping are represented as spheres. Rendered in PyMol from pdb #1CLL; **(B)** Color-coding of residues comprising the inhibitory and CaM-binding domains of CaMKII subunits: Thr 286 (yellow), two K residues shielding the F and N residues (dark green), F (red), N (cyan), initial CaM-binding domain (magenta). **(C)** Ribbon diagram of the atomic structure of an autoinhibited subunit of CaMKII (light green) with the atoms of residues that mask the CaM-trapping amino acids phenylalanine (F, red) and asparagine (N, cyan) shown as light and dark green spheres. Rendered in PyMol from pdb #3SOA; **(D)** Ribbon diagram of “trapped” CaM bound to the extended CaM-binding domain. After autophosphorylation of Thr-286 the inhibitory segment assumes a fully extended conformation. The critical F and N residues are exposed and residues on both the N- and C-termini of CaM (blue) bind tightly around the FNARRK portion of the CaM binding domain. Rendered in PyMol from pdb #2WEL.

In an important study, Meyer et al. (1992) found that the initial binding of Ca^2+^/CaM to a CaMKII subunit occurs with moderate affinity (*K*_D_ = ~50 nM). However, the movement of the inhibitory segment caused by autophosphorylation substantially increases the affinity for Ca^2+^/CaM, a process they referred to as CaM-trapping and Waxham (Putkey and Waxham, 1996) later found that the off-rate of CaM4 is increased from 6.6 s^−1^ to 9×10^−5^ s^−1^, producing a several thousand-fold increase in affinity (*K*_D_ = ~1.8 pM). Tse et al. (2007) showed that autophosphorylation exposes residues Phe293 and Asn294 in the CaM-binding domain, and these residues mediate the high affinity CaM-trapping. After autophosphorylation, residues on both the N- and C-termini of CaM (Figure 2A) bind tightly around the FNARRK sequence in the CaM-binding domain (Figures 1C, 2).

Various investigators have postulated that CaM-trapping leads to an enhanced non-linear increase in the rate of autophosphorylation of CaMKII during high frequency synaptic stimulation (Nicoll and Schulman, 2023; De Koninck and Schulman, 1998; Putney, 1998). However, the tiny spatial compartment of the spine, and the low copy numbers of molecules therein make it impossible to measure enzyme activity experimentally within spine compartments without significantly perturbing the kinetics of protein–protein interactions. Computational models can act as a kind of kinetic “microscope” to explore how detailed dynamics occur within small cellular compartments. They also permit tests of the importance of individual components of a model, by revealing the consequences of the experimental measurements built into it, and of variations of the model’s parameters. Here, we have considered how CaM-trapping will affect the activation and autophosphorylation of CaMKII holoenzymes in a median-sized spine responding to a presynaptic stimulus consisting of two 1 s bursts of 50 Hz presynaptic stimulation, spaced 2 s apart. Under these conditions, we show that CaM-trapping does not influence the proportion of autophosphorylation of CaMKII during the intermediate-strength two-epoch stimulus because it does not increase the number of non-phosphorylated subunits with bound CaM; therefore, it does not increase the probability of new autophosphorylation during the second stimulus epoch. We show that this is the case whether the holoenzymes are distributed uniformly through the spine or are concentrated within the PSD.

Instead, we predict that CaM-trapping will prolong the lifetime of autophosphorylated CaMKII after a stimulus by as much as an order of magnitude, if, as recently proposed, CaM binding to CaMKII sterically hinders binding of phosphatases to autophosphorylated CaMKII, slowing the rate of dephosphorylation (see Pharris et al., 2019). We suggest that *in vitro* experiments with purified proteins to measure the rate of dephosphorylation of autophosphorylated CaMKII by PP1 in the presence and absence of Ca^2+^/CaM will test whether a key effect of CaM-trapping is to slow dephosphorylation of Thr286.

## Methods

### Model development

We modeled activation of CaMKII after a complex presynaptic stimulus delivered to a realistic spine synapse. To do this, we integrated four previous well-tested models to enable us to simulate the activation of dodecameric holoenzymes of CaMKII in a median-sized spine. The models were integrated using the latest version of MCell (MCell4) (Husar et al., 2024) which permits spatial modeling of reactions specified in the efficient BNGL language (Sneddon et al., 2011). The previous models include a model of presynaptic release of glutamate from a CNS synapse (Nadkarni et al., 2010; Nadkarni et al., 2012), a model of Ca^2+^ influx through NMDAR’s and L-type Ca^2+^ channels followed by recovery in reconstructed hippocampal postsynaptic spines and dendrites (Bartol et al., 2015b), a model of activation of CaMKII subunits by Ca^2+^/CaM (Pepke et al., 2010), and a model of autophosphorylation of the dodecameric CaMKII holoenzyme (Ordyan et al., 2020). Each of these models and their parameters were independently validated against experimental data before they were published. As stated in the introduction, the composite model was not constructed to test particular theories, rather it was built to study as closely as possible the behavior that is dictated by actual biological structures. Accordingly, there are essentially no “free variables” that would represent simplifying assumptions. The numbers of molecules and their spatial locations are based upon multiple examples and forms of experimental data, described here and in the original publications. We have not attempted to model here the full range of influences that regulate CaMKII activity in the spine synapse because such an analysis will require several manuscripts of this size; rather we present an initial model that provides first-order quantitative information about how the CaMKII holoenzyme in a spine would respond during the first 10–20 s of activation of NMDARs by a complex stimulus. In future studies, we will be able to add additional elements in a step-by-step fashion, including other CaM-binding proteins (e.g., Ordyan et al., 2020; Gaertner et al., 2004), autophosphorylation at thr305-306 (Colbran and Soderling, 1990; Patton et al., 1990), holoenzymes containing α and β subunits (Bennett et al., 1983; Chien et al., 2024) and interholoenzyme autophosphorylation (Lucic et al., 2023), to determine how they influence the dynamics of activation of CaMKII. In longer term simulations, we will be able to explore binding of CaMKII to downstream proteins, including GluN2B (Tullis et al., 2023; Chen et al., 2024) and cytoskeletal proteins (Walikonis et al., 2001; Robison et al., 2005); and its action on enzymatic targets (e.g., Walkup et al., 2016; Araki et al., 2024). Thus, the model will be a powerful tool to study precise biochemical mechanisms of synaptic plasticity by simulating the exact kinetics of the intricate reactions triggered in the first minutes of Ca^2+^ influx through NMDARs.

### MCell 4 platform

The models are built with the updated agent-based modeling platform MCell4 (Husar et al., 2024) which integrates the rule-based, network-free simulation framework of BioNetGen (Faeder et al., 2009) with the spatial simulation capabilities of MCell (Kerr et al., 2008; Tapia et al., 2019). “Network free” is a term of art referring to the fact that the reaction rules are evaluated on-demand and used directly by the simulator at run time, in contrast to differential equation-based methods and Gillespie-based stochastic methods which require that the rules be expanded into complete reaction networks and stored at the beginning of the simulation. For very large reaction networks, such as the activation network for CaMKII, the savings in computational time and memory usage are extremely large and make computations tractable on high performance computing facilities available today. MCell4 builds on the earlier MCell-R, which employed the reaction methods of NFsim originally developed as a non-spatial simulation platform to read and compute simulations written in the model specification language BNGL (Sneddon et al., 2011). NFsim’s library of functions carries out the graph operations required for efficient network-free simulation in a non-spatial context. MCell-R extended these operations to encompass the spatial context. Thus, it avoids the need to pre-compute a full reaction network and store each possible state in memory. Instead, it executes each rule as needed. The number of rules specified in BNGL is usually much lower than the number of possible reactions in the full network; thus, memory savings are substantial (Tapia et al., 2019). MCell4 improves upon the performance and generality of MCell-R by replacing the NFsim library with a newly developed library of functions, called libBNG, which implements full integration of network-free BNGL directly in MCell4. The computational performance of MCell4 compared to other platforms is documented in a recent publication (Husar et al., 2024). The number of possible reactions of the CaMKII holoenzyme described below would be computationally intractable in other modeling platforms. A Python API was added to MCell4 enabling the model presented here by permitting customizations to the configurations of molecules and reactions that are not easily encoded in CellBlender, the graphical user interface for MCell (Gupta et al., 2018).

### Work flow

The workflow for simulation with the composite models was divided into three parts. In Part 1, the glutamate signals were computed separately for 50 seeds and saved to files. In Part 2, the postsynaptic EPSP and bAP voltages at the spine were computed separately in NEURON and saved to files. For each individual simulation, steps 2 and 3 of Part 2, and all of Part 3 were computed in MCell4 and the changes in state of selected species were saved to output files for analysis and plotting.

PART 1: Glutamate signals generated by electrical stimulation of the presynaptic terminal were simulated according to the model of stochastic, stimulus-driven vesicle fusion (Nadkarni et al., 2010). (1) Digitized waveforms of presynaptic APs were combined to generate the presynaptic stimulus (as in ref. Nadkarni et al., 2010). (2) The timing of vesicle fusion and glutamate release after the presynaptic stimulus was simulated as described in Nadkarni et al. (2010) and the timings for each of 50 random seeds were saved in a file to be used in step 4 and in Part two. (3) The time course of glutamate diffusion after its release into the synaptic cleft was simulated for synapse #37 in the reconstructed neuropil from Bartol et al. (2015b). The shape of this diffusion curve was fit with a function. (4) For each seed, the time-varying glutamate concentration in the cleft was computed from glutamate release times (step 2) and the diffusion function (step 3) and saved to a file.

PART 2: Postsynaptic Ca^2+^ flux through NMDARs and the resulting spine concentrations were simulated in spine #37 from the neuropil model, with a simplified dendritic boundary. (1) The excitatory postsynaptic potential (EPSP), postsynaptic AP, and back-propagating action potential (bAP) resulting from a single stimulus were simulated for a pyramidal neuron, in the program NEURON. The EPSP for a glutamate release event and the resulting bAP at a dendritic spine were stored in a file. (2) The glutamate release times from part one were used to specify the timing of individual EPSPs and the arrival of a bAP 10 msecs later. EPSPs and bAP were summed to obtain the resulting voltage changes at the dendritic spine. (3) Binding of glutamate to each NMDAR was simulated for each seed from the time-varying glutamate concentration in the cleft (Part one). When glutamate binding coincided with depolarization by the bAP, Ca^2+^ flux through the NMDAR was initiated according to the kinetic behavior described by Vargas-Caballero and Robinson and shown in Bartol et al. (2015b).

PART 3: Autophosphorylation of CaMKII holoenzymes was simulated in the spine head, triggered by Ca^2+^ flux through the NMDARs. (1) Binding events among Ca^2+^, calmodulin, and subunits of the CaMKII holoenzyme were simulated within the spine head according to the model in Pepke et al. (2010). (1a) Simulations were performed with and without CaM-trapping. (1b) Simulations were performed with and without localization of CaMKII in a PSD capsule. (2) After CaM binding, autophosphorylation of individual subunits at thr286 in each holoenzyme (see Figure 1B) was simulated according to the model rules described below. (3) Dephosphorylation of thr286 was simulated as a Michaelis–Menten process triggered by interaction of PP1 with CaMKII subunits.

Details for each Part are described below.

### Presynaptic stimulus

**Figure 3:**
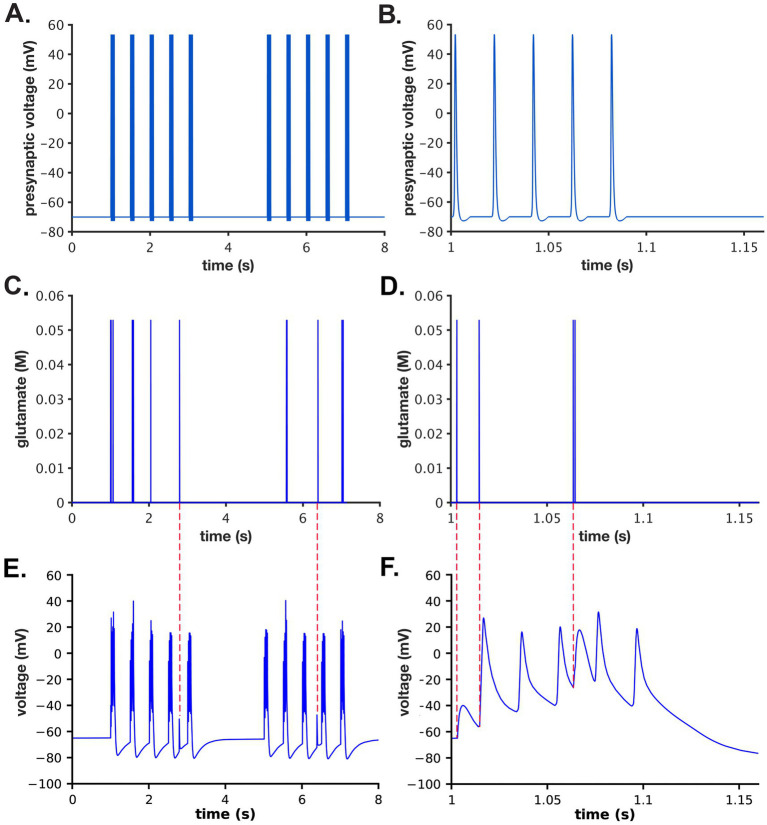
Presynaptic stimulus, glutamate release, and postsynaptic potential. **(A)** A two epoch stimulus of 5 Hz bursts of APs was applied to the presynaptic bouton as described in the text. Each burst was comprised of 5 action potentials delivered at 50 Hz. The stimulus began at 1 s. The two epochs were separated by 2 s. **(B)** Data from **(A)** between 1 and 1.15 s. **(C)** One example (from a single simulation initiated with seed #50, as described in the text) of the 50 different glutamate concentration distributions in the cleft after release from single vesicles during the stimulus shown in **(A)**. **(D)** Data from **(C)** between 1 and 1.15 s. **(E)** Postsynaptic membrane potentials during the single simulation initiated with seed #50. **(F)** Data from **(E)** between 1 and 1.15 s. Note that no glutamate release occurred prior to the third bAP; two release events occurred prior to the fourth bAP; and no glutamate release occurred prior to the fifth bAP. Red dashed lines between **(C,E)** indicate ectopic release events and their associate postsynaptic potentials. Red dashed lines between **(D,F)** indicate EPSPs generated by glutamate release events depicted in **(D)**.

We used a stimulus consisting of two epochs of presynaptic action potentials. Each epoch consisted of five bursts of action potentials (APs) delivered sequentially at 5 Hz (e.g., 5 bursts per sec). Each burst contained 5 action potentials delivered at 50 Hz (e.g., 5 APs per 100 msec). The two epochs were separated by 2 s (Figures 3A,B). The AP waveforms were generated as described in Nadkarni et al. (2010).

### Glutamate release

Glutamate releases from the presynaptic terminal during the stimulus were simulated based upon our previous MCell model of release from presynaptic vesicles (Nadkarni et al., 2010; Nadkarni et al., 2012). The model of release includes 37 measured parameters, adjusted to 34°C. The model was verified against published experimental studies including: (1) the time course of change of release probability after a single AP (Goda and Stevens, 1994); (2) magnitude and time course of paired-pulse facilitation (Dobrunz et al., 1997); and (3) time course of recovery of the release probability after vesicle depletion (Nadkarni et al., 2012).

A set of 50 realistic stochastic timings of release of glutamate from presynaptic vesicles driven by the two-epoch stimulus were computed in MCell initiated by 50 random seeds. The timings of glutamate release computed in this model took into account stochastic short term plasticity of vesicle fusion, including facilitation by residual Ca^2+^ and depression due to depletion of docked vesicles and resulting decreases in release probability. We assumed a median vesicle size containing 1,500 molecules of glutamate (see Figure 6 in Ref. Haas et al., 2018). Releases did not occur with each of the 50 simulated presynaptic APs because for this median-sized spine we simulated the initial release probability as ~0.2. The active zone initially contained 7 docked vesicles. Release was facilitated after the first few stimuli (due to residual presynaptic Ca^2+^ in the active zone) and then depressed due to the reduction in the number of docked vesicles. Simulations initiated by each seed resulted in different stochastic trains of release times. Empty vesicle docking sites were reoccupied with a time constant of 5 s (i.e., 0.2 per second rate of redocking per site).

The precise timing of the Glu release after a presynaptic AP was stochastic and sometimes occurred 10’s of ms after the AP, especially after a long train of stimuli when the [Ca^2+^] in the presynaptic terminal was high (Figures 3C,D). If the vesicle docking sites were highly depleted and [Ca^2+^] was elevated in the terminal, a vesicle sometimes appeared to release “spontaneously” at the moment it docked. The sets of timings of glutamate release were indexed by their seed numbers and stored in files in a directory named “glu_release_times_stp_5at50Hz_5at2Hz_2x” and used in the python script, “glu_dependent_rates.py” embedded in the model.

### Concentration of glutamate in the cleft

The full spatial model of hippocampal neuropil described in Bartol et al. (2015b) was used to measure the time courses of glutamate concentration in the synaptic cleft at the location of the cluster of 15 NMDA receptors (see below). Each vesicle fusion released 1,500 molecules of Glu and the vesicle release sites were assumed to be located over the NMDAR cluster. NMDAR clusters are usually located immediately adjacent to AMPAR clusters (Goncalves et al., 2020), which have been found to be coupled to presynaptic release sites to form transsynaptic “nanocolumns” (Biederer et al., 2017). Placement of release sites directly over the NMDAR cluster will not have a large effect on glutamate binding because the on-rate of glutamate binding to NMDARs is much slower than glutamate’s rate of diffusion (Li et al., 2021). However, placing the release sites directly over the NMDAR cluster means that these simulations did not reflect the small variability in glutamate concentration at NMDARs that would result from stochastic locations of nanocolumns and release sites. To measure the glutamate concentration, a measurement box was positioned in the cleft covering the extracellular domains of the receptors. The box was transparent to the diffusion of glutamate. Its depth was equal to the height of the cleft and the sides of the box enclosed the NMDAR cluster. The time course of concentration of glutamate within the box was recorded for a series of single releases and then averaged. The averaged transient of glutamate in the box was brief with a decay time constant, 𝜏, of about 1.2 μs, and had a peak concentration of ~70 mM. The time-varying glutamate concentration in the cleft in response to the complex stimulus was computed by summing together the individual glutamate transients occurring at each release time. The resulting concentration time course was used to create a pseudo-first order approximation of binding of glutamate to NMDARs, as follows. If k_plus_ is the second order rate constant for glutamate binding to NMDARs, the rate of binding of glutamate is given by [glu] x [NMDAR] x k_plus_. Binding of glutamate to each NMDAR was specified as a first-order transition from an unbound NMDAR to an NMDAR with bound glutamate. The measured time course of the glutamate concentration was used to compute pseudo-first order rate constants, k_plus_^−^effective = [glu] x k_plus_, for each release event and for each seed, as specified in the python script, “glu_dependent_rates.py.” The k_plus_-effective at each time was then used to determine the probability of transition of each NMDAR to the bound state for each seed during the simulation.

### NMDAR response and Ca^2+^ handling in the spine

**Figure 4:**
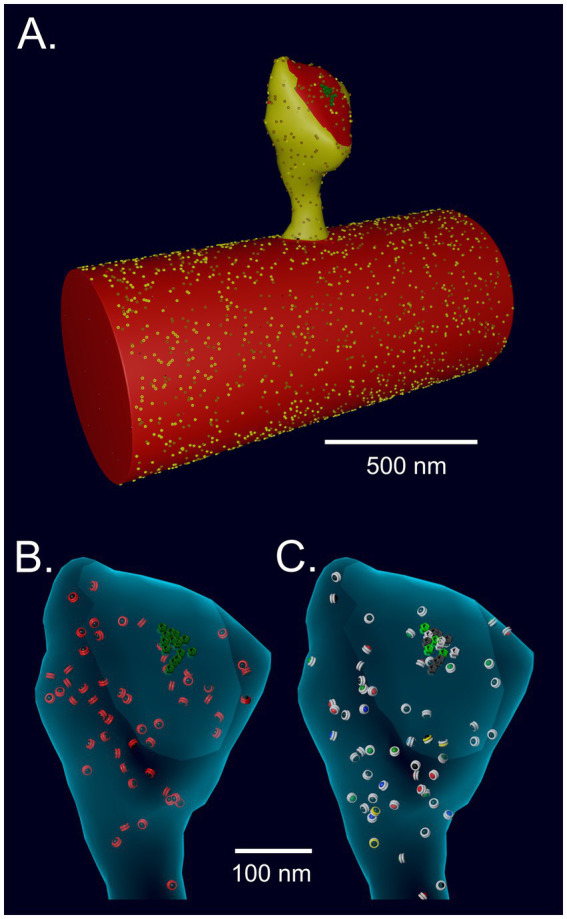
**(A)** Spine # 37 from Bartol et al. (2015b) attached to a cylinder with visible glyphs representing surface proteins. The PSD area on the spine and the attached cylinder are shown in red. **(B)** A translucent view of spine #37 at instantiation showing surface NMDARs (green) and internal inactive CaMKII holoenzymes (red glyphs). **(C)** Spine #37 at *t* = 159 msecs after the start of a stimulus. Open NMDARs on the surface are represented as white glyphs. The outer rings of the CaMKII glyphs have changed color to white indicating where CaM with 4 bound Ca^2+^ ions has bound to the subunits (see Results). The color of the internal spheres in the glyphs indicates the number of autophosphorylated subunits in each ring.

The response of NMDARs was modeled with the kinetics used in our earlier MCell model of Ca^2+^ influx into the spine through NMDARs (Bartol et al., 2015b; Vargas-Caballero and Robinson, 2004). The spine model was originally constructed with MCell3 within the geometry of a hippocampal dendrite reconstructed from serial sections of a 6 μm × 6 μm × 5 μm cube of neuropil imaged by electron microscopy (Bartol et al., 2015b). The model includes all of the major sources and sinks of Ca^2+^ ion known to be present in the membranes and cytosol of spines and dendrites. It incorporates experimentally measured kinetics of Ca^2+^ influx through Ca^2+^ channels and NMDARs, including desensitization and the flickering removal of the Mg^2+^ block by a back-propagating action potential. It also includes experimentally measured parameters of Ca^2+^ buffering, and Ca^2+^ removal from the spine via Ca^2+^ exchangers, pumps, and diffusion. Most of the 85 parameters in the 2015 model were taken from experimental literature; some were derived from the thermodynamic requirement for microscopic reversibility. Sixty nine of the parameters had been measured at 24°C and were adjusted to 34°C. The remaining parameters, for the PMCA and SERCA pumps (16), were used as measured at 37°C. Predicted Ca^2+^ fluxes induced by EPSPs and bAPs simulated in the model were validated against an experimental study by Sabatini et al. (2002). For the model presented here, one cluster of 15 NMDARs (Figure 4) was added near the center of the PSD on the reconstructed spine membrane, reflecting recent data about the number and arrangement of NMDARs in hippocampal synapses (e.g., Goncalves et al., 2020; Racca et al., 2000). The positions of other molecules in the spine are documented in detail in the original publication (Bartol et al., 2015b).

### Spine geometry

To create an accurate model of the response of CaMKII to Ca^2+^ influx inside a spine, while also minimizing computational time, we imported the geometry of a median-sized spine (#37) from the reconstruction in Bartol et al. (2015b) and attached it to a cylinder 0.7 μm in diameter (Sabatini et al., 2002) and 1.5 μm long, created in Blender (Figure 4). The wall of the spine has the shape of the inner membrane leaflet, and is reflective to all the volume molecules. The mesh representing the attachment of the spine to the cylinder was adjusted to make it free of leaks (i.e., “water-tight”). The volume of the cytosol of the spine is 0.016 μm^3^ (0.016 fl) and the PSD diameter is ~200 nm. The cylinder and spine are instantiated with the appropriate numbers of Ca^2+^ pumps and channels (NCX, PMCA, VDCC) in the membrane, and with cytosolic molecules Ca^2+^, calbindin, and immobile buffers, as described for the spine and dendritic shaft in Bartol et al. (2015b). We added CaMKII holoenzymes to the cytosol of the spine, encoded as described below, at a concentration of 6.67 μM (60 holoenzymes); thus, individual subunits were present at a concentration of 80 μM (720 subunits, Bennett et al., 1983; Erondu and Kennedy, 1985). PP1 was added to the spine cytosol at a “baseline” concentration of 1.25 μM (12 molecules), reflecting experimental measurements of the approximate concentration of PPI in neuronal cytosol and its enrichment in spines (Ingebritsen et al., 1983). The k_cat_ for PP1 was set to 11.5/s (Watanabe et al., 2001) and the K_M_ for binding to CaMKII was 11 μM (Bradshaw et al., 2003). Dephosphorylation was modeled as a Michaelis/Menten reaction (see Parameter Table in Supplementary Methods) triggered stochastically by binding of PP1 to a CaMKII subunit. CaM was added to the cytosol of both the spine and the cylinder at a concentration of 30 μM (Watterson et al., 1976; Kakiuchi et al., 1982), which is 290 CaM molecules in the spine and ~ 10,300 in the cylinder. To test the effect of varying the concentration of PP1 on autophosphorylation, the concentration of PP1 in the spine was varied from 0.65 μM to 5 μM. Details of the calculations of initial numbers of CaMKII, CaM, and PP1 are in Supplementary Methods.

### Boundary conditions

In the previous model of Ca^2+^ flux (Bartol et al., 2015b), the spines were attached to a reconstructed dendrite. Diffusing molecules, including Ca^2+^ and calbindin, moved freely through the neck between the spine and the dendritic cytoplasm. The VDCCs, PMCAs, and NCXs in the dendritic membrane contributed to the decay time of the spine Ca^2+^ transient. In that model, we showed that in the first 100 msecs after a stimulus, approximately half of the Ca^2+^ entering spine #37 after an EPSP followed by a bAP exited by diffusion through the neck, either free or bound to calbindin, consistent with (Sabatini et al., 2002). Thus, in the model presented here, Ca^2+^, calmodulin, and calbindin were allowed to move freely between the spine and the cylinder, but CaMKII holoenzymes and PPI were confined to the spine to facilitate simulation and counting of transformations of CaMKII and its dephosphorylation by PP1 in the spine. To implement this, we constructed a “spine shell” consisting of a mesh encasing the spine at a uniform distance of 20 nm from the spine membrane. The shell cuts through the interface at the junction between the spine and the cylinder so that it forms a closed barrier at the base of the spine neck. CaMKII holoenzymes and PP1 are confined to the spine by making this shell reflective to CaMKII and PP1, but transparent to diffusing Ca^2+^, calmodulin, and calbindin. The dynamics of Ca^2+^, CaM, and CaMKII are quantified within the spine shell.

The length of the cylinder was enlarged until the results of simulations of activation of CaMKII within the spine converged when we enlarged it further. Convergence was achieved between cylinder lengths of 1.5 and 1.8 μm. Therefore, the length of the cylinder was set at 1.5 μm, ensuring that the cylinder provides a boundary equivalent to the dendritic shaft.

### Calmodulin

The model of initial binding interactions among Ca^2+^, calmodulin, and individual subunits of CaMKII was based upon our previous non-spatial model of the dynamics of activation of monomeric CaMKII subunits by Ca^2+^/CaM as the concentration of Ca^2+^ rises (Pepke et al., 2010). This model was created in Mathematica and includes states of Ca^2+^/CaM with one, two, three, or four bound Ca^2+^ ions. The 55 parameters for Ca^2+^ binding to free CaM and for Ca^2+^ binding to CaM bound to CaMKII were measured experimentally at 30°C by various investigators, including the Kennedy lab (Shifman et al., 2006), or were calculated from the measured parameters using the thermodynamic requirement for microscopic reversibility, involving “detailed balance” of ∆G within reaction loops (see Pepke et al., 2010).

In the present model, the CaM molecule was encoded in BNGL as follows: CaM (C ~ 0 ~ 1 ~ 2, N ~ 0 ~ 1 ~ 2, camkii). In BNGL syntax, the name of the molecule is followed in parentheses by a set of “components” demarcated by ~, which represent binding sites or modification sites. As shown in Figure 2A, CaM has four binding sites for Ca^2+^, two at the C-terminus and two at the N-terminus; thus, the three states for each terminus (~0,~1, or ~ 2 of components C or N) represent zero bound Ca^2+^, one bound Ca^2+^, or two bound Ca^2+^, respectively. The camkii component is the binding site for a CaMKII subunit. The model in Pepke et al. (2010) did not include CaM trapping following autophosphorylation; CaM-trapping was introduced into the model of the holoenzyme as described below.

### CaMKII holoenzyme

We updated the model of CaMKII subunits within a dodecameric holoenzyme from Ordyan et al. (2020). This non-spatial model employed BioNetGen language (BNGL) and the simulation algorithm NFsim, to build the structure of the holoenzyme, simulate the kinetics of interaction of its subunits with Ca^2+^/CaM, and simulate the subsequent autophosphorylation and dephosphorylation by phosphatase of individual subunits. Parameters and reactions of subunits in this model were based upon those in Pepke et al. (2010), which have been repeatedly vetted for accuracy. This model did not include CaM-trapping or spatial dynamics and was originally used to examine the potential influence of neurogranin on integration and summation of autophosphorylation following increases in Ca^2+^ concentration.

**Figure 5:**
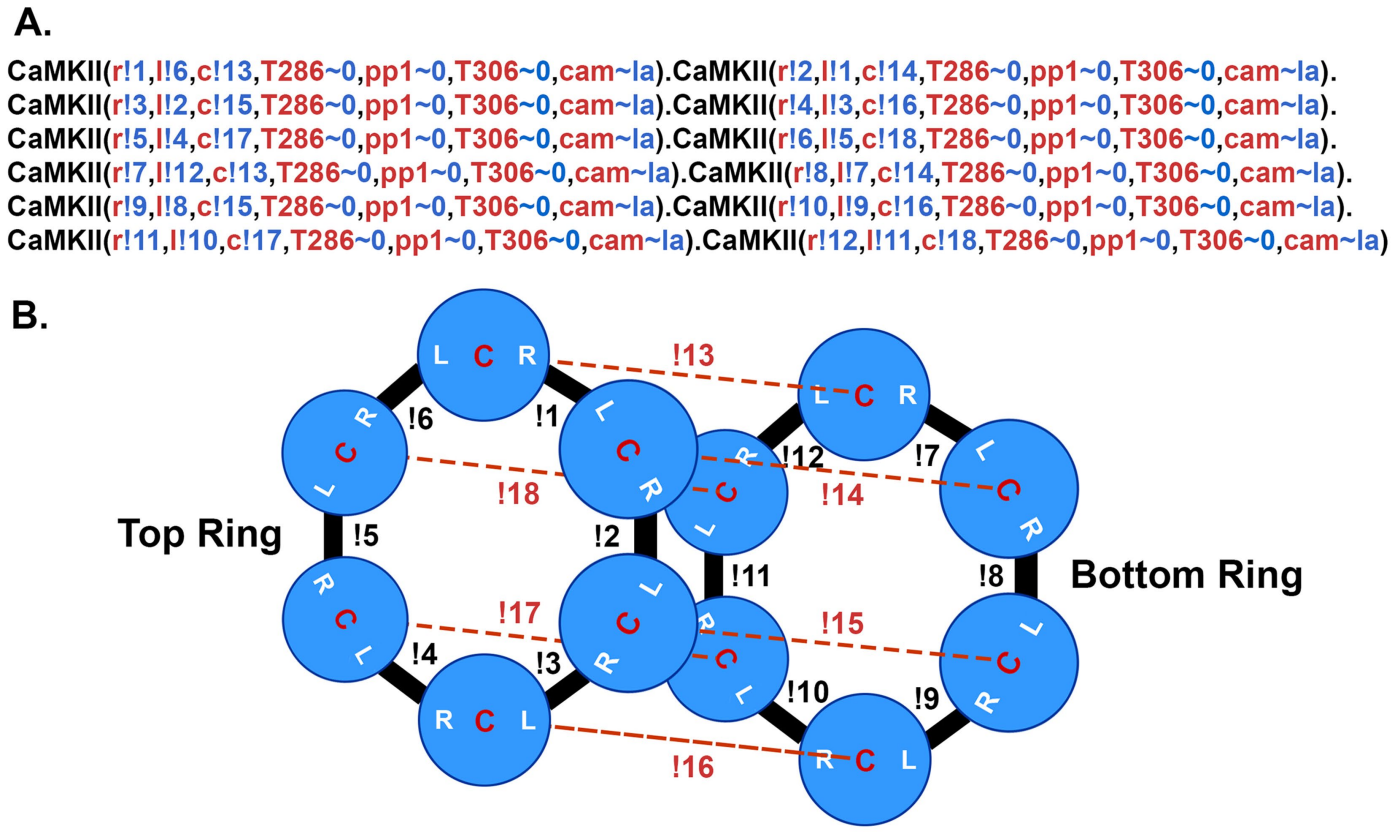
BNGL model of the CaMKII holoenzyme. **(A)** The BNGL code represents a CaMKII holoenzyme as it is initialized in a simulation. A single subunit is defined in BNGL as follows (see Ordyan et al., 2020): CaMKII (r,l,c,T286 ~ 0 ~ P, pp1 ~ 0 ~ 1,T306 ~ 0 ~ P,cam ~ la ~ ha). The holoenzyme is coded as 12 connected subunits. Components r, l, and c represent binding sites among subunits in the holoenzyme as diagrammed in **(B)**. T286 is a component representing the autophosphorylation site threonine-286 which can be present in one of two states ~0 (unphosphorylated) or ~ P (phosphorylated). Pp1 is a site for binding of protein phosphatase-1 which can be either unbound (~0) or bound to PP-1 (~1). T306 is the autophosphorylation site threonine-306. Cam represents the binding site for Ca^2+^/CaM which is allowed to be in either a low affinity binding-state (~la) with *K*_D_ = 65 nM or the high affinity state associated with CaM-trapping (~ha) with *K*_D_ = 1.8 pM. Bonded subunits are separated by a period. Bonds between components of subunits are represented by the symbol! (bang) followed by a number. The number must agree for the two components that are bound to each other. The holoenzyme is initialized with no sites phosphorylated and all cam components in the ~la state. **(B)** Diagram of the bonds formed among the r, l, and c sites on each subunit to encode the dodecameric holoenzyme.

In the present, updated model, the individual subunits are specified in BNGL as follows: <CaMKII (l, r, c, T286 ~ 0 ~ P, pp1 ~ 0 ~ 1, T306 ~ 0 ~ P, cam ~ la ~ ha) > (Figure 5A). Components l, r, and c are sites of interaction within the holoenzyme; l is a binding site at the left side of the subunit, r at the right side of the subunit, and c (center) a site that interacts with a subunit in the opposite six-membered ring (Figure 5B). The unphosphorylated subunits were added (“instantiated”) into the model by specifying irreversible binding at these three sites (Figure 5) to comprise fully specified CaMKII holoenzymes. In BNGL syntax, a bond between two molecules is represented as a period and the components involved in binding are followed by a! (pronounced “bang”) and a number. For example a bond between the left side of one subunit and the right side of another is represented as CaMKII (l!1, r, c, T286 ~ 0, pp1 ~ 0, T306 ~ 0, cam ~ la), CaMKII (l, r!1, c, T286 ~ 0, pp1 ~ 0, T306 ~ 0, cam ~ la). The! can be followed by any number so long as the two binding components have the same number. “T286” is the threonine autophosphorylation site that confers Ca^2+^-independence; it is modeled as having two states, either unphosphorylated (~0) or phosphorylated (~P). pp1 is a binding site for the catalytic subunit of protein phosphatase-1; it can have two states, either unbound (~0) or bound (~1) to PP1. “T306” is the threonine autophosphorylation site that can be phosphorylated after CaM unbinds from the subunit. The T306 site remained unphosphorylated (~0) in the present study. The “cam” component is the binding site for calmodulin; it can have two states, either low affinity (~la), or high affinity (~ha). The ~ha state is the high affinity state that appears when a subunit is autophosphorylated at T286; i.e., the “CaM-trapping” state (see reaction rules below).

### Reaction rules

Reaction rules are specified in BNGL as discrete transformations. For example, the rule “CaM(C ~ 0,camkii) + ca ↔ CaM(C ~ 1,camkii) k_on1C, k_off1C” encodes reversible binding of one Ca^2+^ to the C-terminus of CaM for CaM not bound to CaMKII with k_on_ = k_on1C and k_off_ = k_off1C. The rate constants for each rule are used to compute the probability that the transformation occurs when the two molecules involved in a bimolecular transformation collide. Unimolecular transformations occur at a future time step obtained by sampling the expected first-order decay time course given by the rate constant. At run time, a random number generator is initialized with a seed number for each individual simulation that specifies a sequence of random numbers that are used to generate the sequence of Brownian motion random-walk trajectories and the probabilities of state transitions for the simulation.

As described above, the model of binding among Ca^2+^, CaM, and CaMKII subunits in the holoenzyme includes rules for binding/unbinding of Ca^2+^ to each of the four sites on CaM when CaM is not bound to CaMKII, binding/unbinding of each state of CaM to a subunit of CaMKII in the holoenzyme, and binding/unbinding of Ca^2+^ to each of the four sites on CaM when CaM is bound to CaMKII. Binding of CaM to CaMKII increases its affinity for Ca^2+^, as expected from thermodynamic detailed balance. These rules and experimental parameters (as measured at 25°C) are taken from the model in Pepke et al. (2010).

Rules are included for autophosphorylation of one subunit of CaMKII by another. Autophos phorylation at Thr 286 occurs between adjacent subunits in a holoenzyme when they were both active, either because they both have bound Ca^2+^/CaM or because the “right-hand” subunit is already autophosphorylated. We modeled the autophosphorylation reaction as proceeding in one direction around a ring, with the “right-hand” subunit acting as the kinase and the “left-hand” subunit acting as substrate. This situation fits the structural analysis of subunit interactions during autophosphorylation (Rellos et al., 2010). Parameters that govern the probability of autophosphorylation were derived from measurements in Shifman et al. (2006). In that study autophosphorylation rates (turnover numbers) were measured at 30°C with a temperature controlled quench-flow device for CaMKII bound to wild-type CaM4, and bound to mutant CaM’s that bind Ca^2+^ only at the N or C termini (Shifman et al., 2006). We generated 64 permutations of the autophosphorylation rules between neighboring CaMx-bound subunits in a holoenzyme (CaMx denotes CaM with all possible numbers of bound Ca^2+^ ions). Pairs of subunits, both of which contain bound CaM4, undergo autophosphorylation of the left subunit at the rate k_pCaM4 (0.96 s^−1^, Shifman et al., 2006). All other permutations underwent autophosphorylation of the left subunit at the rate k_pCaMpartial (0.1 s^−1^), which is a coarse-grained approximation of the rates measured by Shifman et al. (2006). Eight rules are included in which the right-hand subunit of a pair is autophosphorylated but no longer has bound CaM and the left hand subunit contains bound CaMx. For these rules, when the target subunit is bound to CaM4, the rate of autophosphorylation is k_pCaM4. When the target subunit was bound to CaM with less than 4 Ca^2+^ ions bound, the rate of autophosphorylation is k_pCaMpartial.

### CaM-trapping

Meyer et al. (1992) showed that the initial binding of Ca^2+^/CaM to a subunit within the CaMKII holoenzyme occurs with moderate affinity (*K*_D_ = ~50 nM). However, following autophosphorylation, the inhibitory domain is moved entirely away from the substrate pocket (Rellos et al., 2010), leading to an ~1,000-fold higher affinity for CaM, referred to as CaM-trapping (see Introduction, Meyer et al., 1992; Putkey and Waxham, 1996; Tse et al., 2007). We added reactions to the model to simulate CaM-trapping in order to enable comparison of simulations with and without CaM-trapping. The k_on for CaM4 prior to trapping (measured at 30°C) was taken from Meyer et al. (1992), and the prolonged k_off for CaM4 in the trapped state (measured at 22°C) was taken from Putkey and Waxham (1996). A component state termed cam ~ la was added to CaMKII subunits representing the initial lower affinity CaM-binding site; the component state cam ~ ha was added representing the higher affinity site present following autophosphorylation of a subunit. The off rates of individual CaMx species from CaMKII for each state were calculated based on the necessity that ∆G = 0 around a reaction cycle (“detailed balance,” e.g., see Pepke et al., 2010). We set the rate of transition from cam ~ la to cam ~ ha following autophosphorylation equal to the rate of dissociation of CaMx from the cam ~ la site.

The reactions of CaMKII holoenzymes and CaM and the autophosphorylation reactions were implemented within the spine geometry shown in Figure 4, which was taken from the model of Ca^2+^ flux described above (Bartol et al., 2015b). The inclusion of reactions leading to CaM-trapping by autophosphorylated CaMKII subunits enabled comparison of activation and autophosphorylation kinetics in the presence and absence of CaM-trapping. Inclusion of reactions specifying dephosphorylation by PP1 enabled examination of the effects of variations in the amounts of PP1 activity. Finally, the creation of a version of the model specifying that dephosphorylation by PP1 does not occur when CaM is bound to an autophosphorylated subunit enabled comparison of the kinetics of autophosphorylation with and without blocking of dephosphorylation by bound CaM.

### Postsynaptic EPSPs and action potentials

A single 25 mV postsynaptic EPSP resulting from release of glutamate from one vesicle activating both AMPA and NMDA receptors was simulated in the program NEURON. The 25 mV peak was the average size for hippocampal CA1 pyramidal neurons, calculated by Harnett et al. (2012). Because the NEURON model did not contain glutamate receptors, the excitatory postsynaptic potential (EPSP) was simulated by injecting current into the spine head as an alpha function such that a 25 mV peak EPSP was produced. The peak of the EPSP occurred about 3 ms after release and decayed with a 𝜏 of ~10 ms. The time course of the EPSP was saved to the data file “post_epsp_spine_voltage.dat” to be used at runtime.

The APs in the soma and the associated back-propagating APs (bAPs) in the dendrites arising from the two-epoch stimulus were simulated in the program NEURON by injecting current into the axon hillock of pyramidal neuron, model “j4” (Mainen et al., 1995). The voltages experienced at a spine located on a dendritic branch ∼100 μm from the soma were recorded, as described in Keller et al. (2008) and Bartol et al. (2015b). The spine was chosen so that the bAP arrived at the spine 10 ms after the presynaptic AP. The current injection was large enough to reliably initiate an AP in the soma and subsequent bAP, as it would if applied to an axon bundle with many synapses ending on the neuron. The ion channels included in the j4 simulation are described more fully in Bartol et al. (2015b) and in the NEURON ModelDB.^1^ The time course of the bAP arriving at the spine was saved to the data file “post_bAP_spine_voltage.dat.”

A python script (vm_dependent_rates_post.py) was written to add together the changes in membrane potential in the spine resulting from EPSPs during simulations for each seed, and from the bAP arriving 10 msecs after the AP in the soma. We assumed that the EPSP and bAP voltages in the spine head summed together linearly. There was a reliable 10 ms delay between the generation of the presynaptic spike in the soma and the arrival of the postsynaptic bAP at our single spine in simulations for each seed. In contrast, the release of glutamate and the resulting timing of the EPSP were more variable because of the biologically realistic stochastic elements built into the simulation of release (Figure 3E). When glutamate was released, it was most often at 10 ms before the arrival of the bAP. However, occasionally, for the reasons discussed above, glutamate release and the associated EPSP did not appear causally linked to the bAP. Two such events are shown at ~2.4 and ~ 6.2 s in Figures 3C,E, which depict results of a single simulation for seed #50. NMDARs open and flux Ca^2+^ into the spine only when they bind glutamate at the same time that the EPSP and bAP depolarize the spine. The coincidence of these events opens the channel and relieves the Mg^2+^ block that prevents movement of Ca^2+^ through the channel (Vargas-Caballero and Robinson, 2004; Ascher and Nowak, 1988). The complex kinetics of the transitions that allow Ca^2+^ flux through the NMDARs are explained in detail in Bartol et al. (2015b).

### Data analysis

The simulations were performed on an HPC cluster at Caltech on 50 nodes each having at least 256 Gbytes of RAM. Monte Carlo simulations in MCell4 (Husar et al., 2024) rely on generation of random numbers to determine the diffusion trajectories and probabilities of each reaction. Each simulation was initiated with a different seed for the random-number generator. Simulation of 50 seeds for each condition, for 11 s of simulated time, required 7 to 10 days of compute time. Output, consisting of numbers of molecular states across time, were stored as ASCII data files. MCell permits output of a “checkpoint file,” that can be used to extend the time of a simulation beyond our standard 11 s if desired. The number of individual simulations for each condition required to obtain high confidence averages was determined by comparing the mean and standard deviation of 30, 40, and 50 simulations of calcium dynamics. Our analyses showed that there was no statistically measurable difference between the population averages for 40 and 50 seeds. We thus used 50 seeds for each condition in our simulation to ensure that we would obtain a full representation of all possible stochastic trajectories. All data are plotted as the mean of 50 seeds.

Output was plotted in MatPlotLib^2^ or Prism (GraphPad).^3^

The time constant (𝛕) of exponential decay of pCaMKII caused by dephosphorylation by PP1 was calculated by fitting the amplitude and decay time of averaged curves of pCaMKII with an equation for a single exponential.

### Materials availability

The supplementary movie, a readme file, and the eight models used to generate the data reported here are available for download as a compressed file at: http://www.mcell.cnl.salk.edu/models/spatial-model-of-CaMKII-2024-1/. They are also available on Zenodo at: https://doi.org/10.5281/zenodo.12764450. MCell4 is available for download at: http://mcell.org/ and as source code at: http://github.com/mcellteam/mcell.

## Results

**Figure 6:**
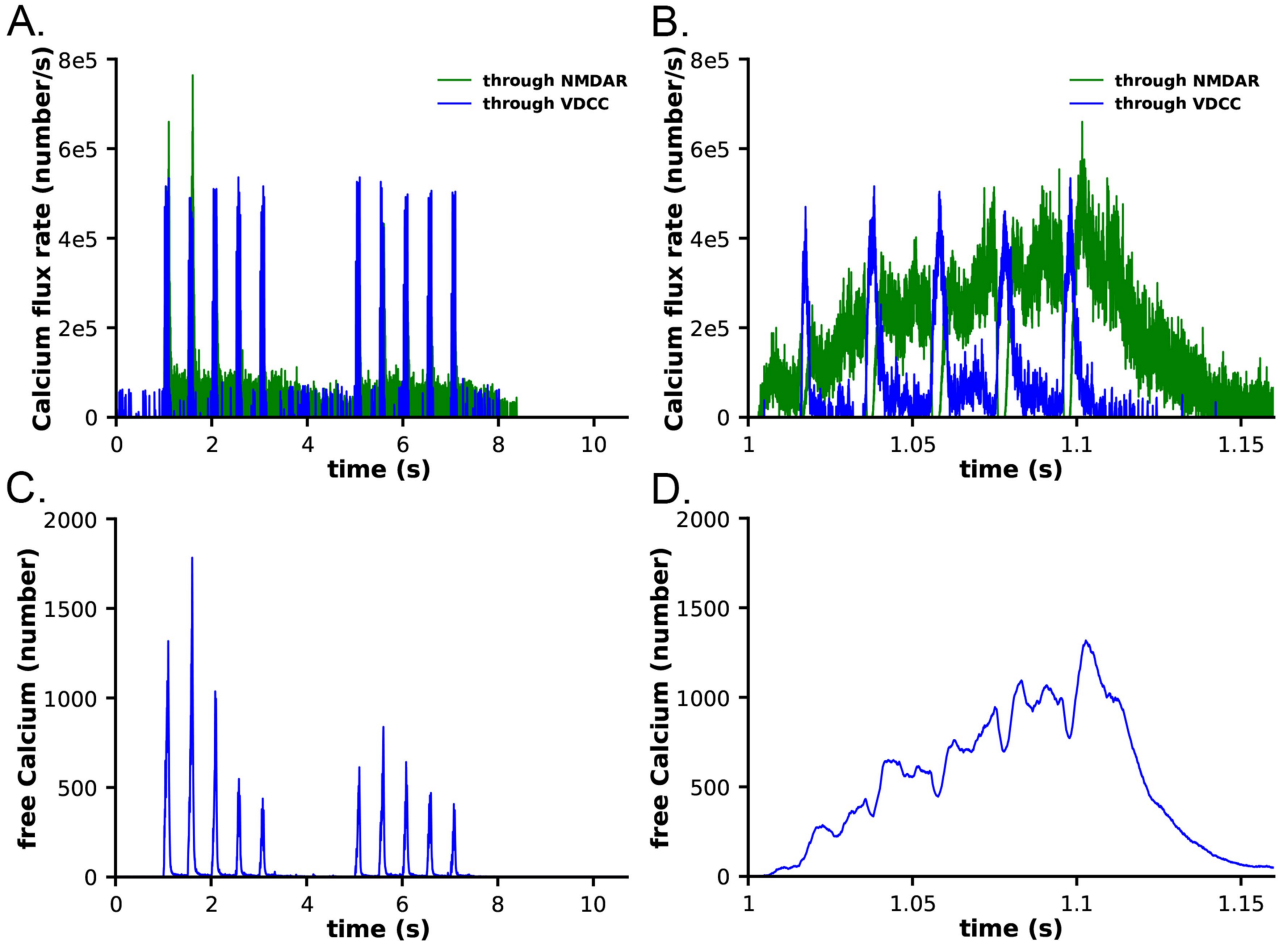
Calcium influx in spines in response to the stimulus shown in Figure 3. **(A)** Calcium flux rate through NMDARs (green) and VDCC (blue). **(B)** Data from **(A)** between 1 and 1.15 s. **(C)** Number of free calcium ions in the spine. **(D)** Data from **(C)** between 1 and 1.15 s. Each curve is the average of 50 individual seeds. The dynamics of NMDAR, VDCC, and calcium are the same in the CaM-trapping and non-trapping models. The dynamics of the different CaM species are shown in Supplementary Figure S2.

Calcium dynamics in the spine in response to the two-epoch stimulus. Ca2+ influx into the spine through the NMDARs and VDCCs was evaluated in response to the two-epoch stimulus shown in Figure 3. As in our previous study (Bartol et al., 2015b), the model captured the complex, stochastic kinetics of NMDAR and VDCC channel openings (Supplementary Figure S1). The NMDAR channel was opened by binding of glutamate, and the Mg^2+^ block of Ca^2+^ flux was relieved by depolarization of the membrane during the bAP (Supplementary Figures S1A,B). During the first epoch, the number of open NMDAR channels decreased for the later three bursts of APs, reflecting the decrease in the number of vesicles releasing glutamate (Figure 3C). The bAPs continued to relieve the Mg^2+^ block for all open NMDAR channels (Supplementary Figures S1A,B). The number of open NMDAR channels decreased substantially for the second epoch due to the lower release probability from the presynaptic terminal caused by short term depression (Figure 3C) and a small effect of NMDAR desensitization. The stimulus caused voltage-dependent openings of the single VDCC in the spine (Supplementary Figures S1C,D). As expected, the stimulus resulted in calcium influx into the spine through NMDARs and VDCCs (Figure 6). Fluxes through NMDARs and VDCCs corresponded to the channel opening dynamics shown in Supplementary Figure S1. Although the peak Ca^2+^ flux through the VDCC was greater than that through the NMDARs, the integrated flux through NMDARs was considerably greater than the integrated flux through the VDCC, as is evident in Figure 6B. As a result of these fluxes, the free Ca^2+^ in the spine increased in response to the stimulus (Figures 6C,D), as did all of the Ca^2+^-bound species of free CaM (Supplementary Figure S2). Total free Ca^2+^ entering during the first epoch was greater than that during the second epoch due to synaptic depression. In this model, CaM can diffuse from the shaft into the spine during the stimulus; thus, the total Ca^2+^-bound CaM and CaM bound to CaMKII exceeded the amount of CaM initially added to the spine alone.

### Sensitivity to protein phosphatase 1 activity

**Figure 7:**
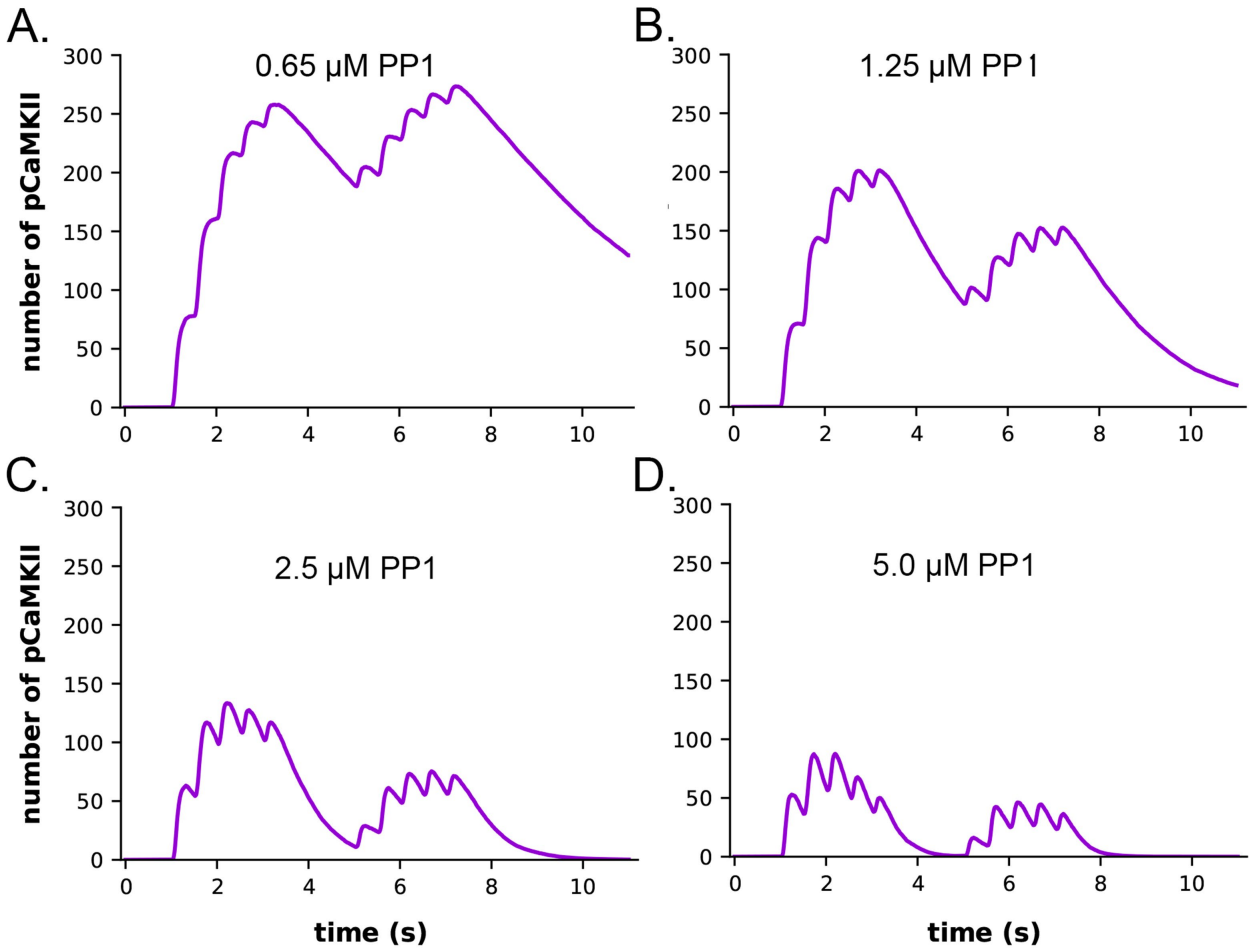
Dynamics of formation of autophosphorylated CaMKII (pCaMKII) subunits in the presence of increasing concentrations of active PP1. **(A)** 0.65 μM PP1 **(B)** 1.25 μM PP1. **(C)** 2.5 μM PP1. **(D)** 5.0 μM PP1 Each curve is the average of 50 simulations run with different random seeds.

It has been shown experimentally that the dynamics of autophosphorylation of CaMKII and induction of LTP are highly sensitive to the concentration of active phosphatase (Strack et al., 1997; Blitzer et al., 1998; Foley et al., 2021). Most of the simulations in this study were obtained with a uniform concentration of active PP1 (1.25 μM) and of CaMKII (80 μM subunits) throughout the spine. We chose this amount of PP1 as a likely median concentration based on the biochemical measurements of Ingebritsen et al. (1983). The PP1 catalytic subunit is regulated and localized *in vivo* by a variety of specialized regulatory subunits (Foley et al., 2021; Bollen et al., 2010), including in synapses by neurabin (Foley et al., 2021; McAvoy et al., 1999) and spinophilin (Allen et al., 1997). Thus, the level of PP1 activity in synaptic spines is likely to be variable and may be regulated by synaptic activity. To test whether the composite model reproduces the observed sensitivity of autophosphorylation to phosphatase activity, we simulated autophosphorylation of CaMKII, varying the concentration of active PP1 in the spine from 0.65 μM to 5 μM (Figure 7). The amount of formation of pCaMKII was extremely sensitive to the level of PP1 (Figure 7). Thus, the composite model reproduces PP1 sensitivity *a priori* within the range of the experimentally measured physiological levels of CaMKII and PPI that we used in the model. It is notable that the numbers and activity of PP1 in the models were not adjusted to confer sensitivity to PP1, rather they were set according to biochemical measurements in the experimental literature. Thus, the model reproduces the sensitivity of induction of LTP to the level of phosphatase activity observed experimentally (Brown et al., 2000; Blitzer et al., 1998).

The data also provide quantitative detail about how the interplay between activation or inhibition of PP1 can exert exquisitely tight control of the progression of activation of CaMKII by a given stimulus. When the amount of active PP1 was reduced by half from 1.25 μM to 0.65 μM, the number of autophosphorylated subunits (pCaMKII) remaining at 5 min after the beginning of the stimulus, which was the time of initiation of the second epoch, was more than doubled (from 90 to 192) (Figure 7). The number of active PP1 molecules bound to a CaMKII subunit at the peak of the first epoch was nearly linearly dependent on the concentration of PP1 between 0.65 and 1.25 μM (Supplementary Figure S3). In the lowest concentration of PP1 (0.65 μM), the residual number of pCaMKII remaining after the first epoch [~190 out of a total of 720 subunits (25%)] was sufficient to support an absolute increase in pCaMKII during the second epoch despite the reduced influx of Ca^2+^ (see Figure 6C). With 25% of subunits autophosphorylated, the average number of pCaMKII subunits in a six-membered ring would be ~1.5. In contrast, In 1.25 μM PP1, the average number of pCaMKII was ~90 out of 720 or ~ 12.5%, with an average of ~0.75 pCaMKII in a six-membered ring. To become newly autophosphorylated, a subunit with newly bound CaMx needs to be located next to an activated subunit in the clockwise direction. A decrease in active PP1 from 1.25 μM to 0.65 μM, increases the probability of this occurring from ~0.75/5.25 (14%) to ~1.5/4.5 (33%). This higher probability increases the likelihood that a subunit with newly bound CaMx will be able to be autophosphorylated by its neighbor.

### Dependence of the dynamics of CaM binding to CaMKII on CaM-trapping

**Figure 8:**
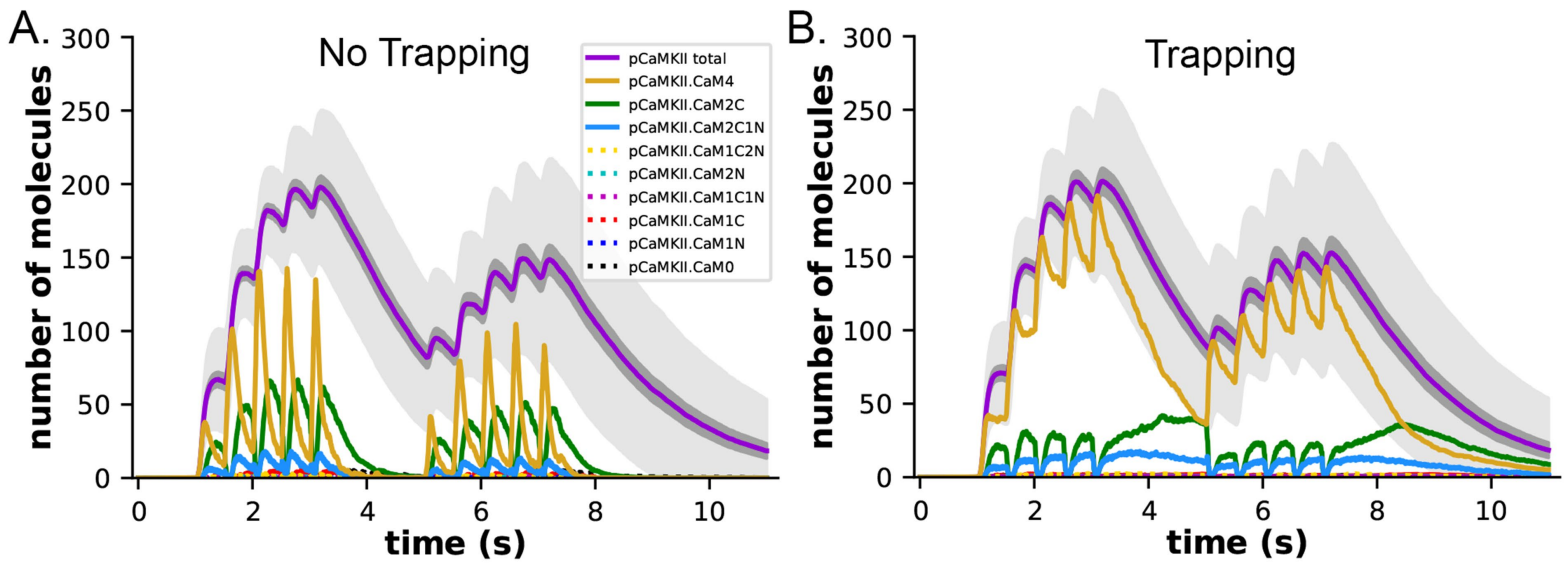
Dynamics of formation of phosphorylated subunits of CaMKII (pCaMKII) and binding of CaM species to them in the presence and absence of CaM-trapping. **(A)** Binding of calcium-bound states of CaM to pCaMKII in the absence of CaM-trapping. Colors are as in the legend. Species shown with dotted lines in the legend were tracked, but numbers of each were 10 or less. **(B)** Binding of calcium-bound states of CaM to pCaMKII in the presence of CaM-trapping. Each curve is the average of 50 simulations initiated by different seeds. The darker gray shading surrounding the curves depicting formation of pCaMKII (magenta) indicates +/− s.e.m. and reflects the accuracy of the average. The lighter gray shading surrounding the curves depicting formation of pCaMKII indicates +/− s.d. The large standard deviation results from the number of stochastic reactions in the model and reflects the biological reality of coupled reactions occurring among a limited number of molecules in a small cellular space. See Supplementary Material for a short movie of a simulation with glyphs as shown in Figures 4B,C.

We measured the dynamics of formation of phosphorylated CaMKII subunits (pCaMKII) in the absence (Figure 8A) and presence (Figure 8B) of CaM-trapping. Each phosphorylated CaMKII subunit could be bound to CaM in any one of eight states; CaM with no bound Ca^2+^ (CaM0), or CaM with 1 to 4 bound Ca^2+^ ‘s (CaM1C, CaM2C, CaM1C1N, CaM2C1N, CaM2N, CaM1C2N, and CaM4). The results show that the total accumulation of pCaMKII was independent of the presence of CaM-trapping with the stimulus applied here. In the presence or absence of CaM-trapping, the numbers of pCaMKII subunits bound to CaM0, CaM1C, CaM1N, CaM2N, CaM1C1N, and CaM1C2N were negligible. In the presence of CaM-trapping, binding of CaM4, CaM2C, and CaM2C1N to pCaMKII was substantially prolonged, and the numbers of these bound CaM species did not fall to zero before the onset of the second epoch (Figure 8B). However, because these species are bound to CaMKII subunits that are already autophosphorylated, the prolonged binding did not increase the likelihood that CaM newly bound during the second epoch would be located next to an active subunit. The presence of trapping did not significantly increase the lifetime of CaM species bound to non-phosphorylated subunits (Supplementary Figure S4). For this reason, CaM-trapping did not increase the number of autophosphorylated subunits produced by the stimulus.

Figures 8A,B show the small s.e.m. of formation of pCaMKII averaged over 50 seeds (darker gray shading), indicating the high accuracy of our calculations of the mean pCaMKII. In contrast, the large s.d. of pCaMKII (lighter gray shading) captures the large dispersion of data that arose because each seed produces a distinct trajectory of stochastic binding and autophosphorylation. The dispersion is the result of the many probabilistic steps involved in formation of pCaMKII upon Ca^2+^ entry. This “noisiness” reflects the actual physiological variability expected for activation of CaMKII in a median-sized synapse by the stimulus that we used. All simulations results shown are averages of 50 independent simulations run with 50 different random seeds and have similar small s.e.m.’s.

### The effect of localization of CaMKII in the PSD on the dynamics of autophosphorylation

**Figure 9:**
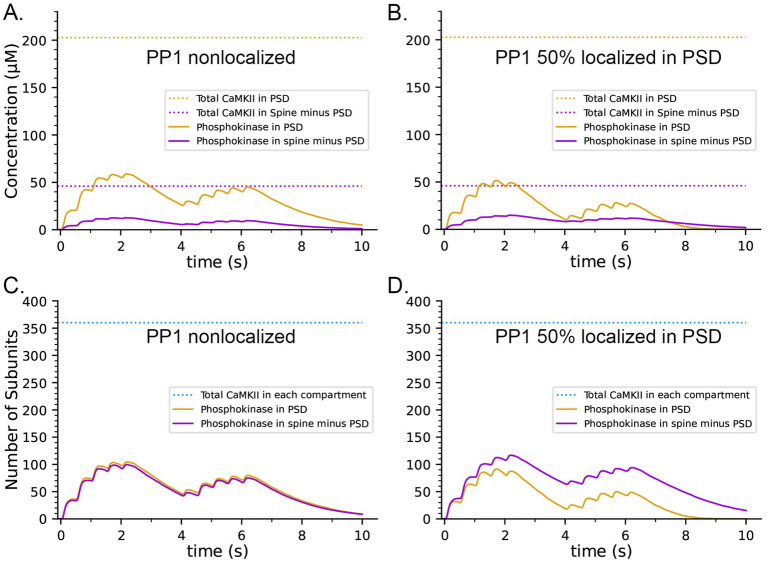
Effects on the dynamics of formation of phosphorylated subunits of CaMKII (pCaMKII) of localization of 50% of CaMKII in the PSD and of 50% of PP1 in the PSD. Lines and colors are as in the legends. **(A)** Concentration of pCaMKII in the PSD capsule and in the rest of the spine (minus the PSD capsule) compared to the total CaMKII in each compartment. **(B)** Same as in **(A)**, but with 50% of PP1 localized in the capsule. **(C)** Numbers of pCaMKII in the PSD capsule and in the rest of the spine, compared to the total CaMKII in each compartment. **(D)** Same as in **(C)**, but with 50% of PP1 localized in the capsule. Curves are averages of 50 simulations initiated with 50 different random seeds.

The concentration of CaMKII in the spine cytosol is high; however *in vivo*, a substantial portion of spine CaMKII is further localized to the PSD as shown by immunofluorescence and biochemical studies (Kennedy et al., 1983; Shen and Meyer, 1999; Shen et al., 2000; Sheng and Hoogenraad, 2007), where it is concentrated within ~50 nm of the postsynaptic membrane (Valtschanoff and Weinberg, 2001). It has been suggested that this portion of CaMKII may be more highly activated and autophosphorylated than cytosolic CaMKII during synaptic stimuli that activate NMDARs (Lisman et al., 2002). To test this idea, we created a capsule associated with the postsynaptic membrane. The capsule has lateral boundaries contiguous with the reconstructed PSD and extends 50 nm into the cytoplasm from the inner membrane leaflet (Valtschanoff and Weinberg, 2001). The volume of the capsule is 0.002915 fL which is ~20% of the spine volume. The walls of the capsule were made reflective to CaMKII and transparent to all other volume molecules. To simulate 50% localization of CaMKII in the PSD, we placed 30 holoenzymes within the PSD capsule (subunit concentration ~ 200 μM) and 30 holoenzymes in the spine cytosol outside the PSD capsule (subunit concentration ~ 43 μM)., The other variables in this model were identical to those in the simulations shown in Figures 8B,D, which included CaM-trapping. Results of simulations with this model are shown in Figures 9A,C. Localization of CaMKII in the PSD has only a minimal effect on the proportion of subunits autophosphorylated (Figure 9C), indicating a small enhancement of autophosphorylation likely arising from the proximity of subunits in the PSD to Ca^2+^ coming through the NMDA receptor. In contrast, as expected, the concentration of pCaMKII in the PSD is approximately 5-fold higher than in the rest of the spine (Figure 9A). Note that the increased concentration of CaMKII in the PSD does not alter the rate of autophosphorylation upon CaM binding because we have modeled autophosphorylation as primarily an intraholoenzyme reaction at this time scale. The higher concentration of CaMKII would, however, enhance the rate of binding of pCaMKII to the nearby GluN2B subunits of the NMDARs (Tullis et al., 2023; Bayer et al., 2001) and to densin-180 located in the PSD (Walikonis et al., 2001; Carlisle et al., 2011). It would increase the rate of interholoenzyme autophosphorylation (Lucic et al., 2023) and the rate of phosphorylation of protein substrates located in the PSD.

It has been reported that pCaMKII is preferentially dephosphorylated by PP1 localized in the PSD (Strack et al., 1997; Foley et al., 2021). To test the effect of localization of PP1 in the PSD, we further modified the model to make the capsule reflective to both CaMKII and PP1; and placed 50% of CaMKII and of PP1 (6 molecules, concentration ~ 3.3 μM) inside the capsule leaving the other 50% in the rest of the spine cytosol (concentration ~ 0.7 μM). Simulations with this modified model revealed that differential localization of PP1 in the PSD decreased formation of pCaMKII in the PSD and enhanced formation of pCaMKII outside the PSD (Figures 9B,D).

### CaM-trapping may influence the rate of dephosphorylation of pCaMKII by prolonging the ability of bound CaM to inhibit binding of PP1

The presence of CaM-trapping did not influence the rate or extent of formation of pCaMKII during the two epoch stimulus that we used here (Compare Figures 8A,B). However, it did significantly prolong the lifetimes of binding of CaM4, CaM2C, and CaM2C1N to CaMKII (Figures 8B,D). In an earlier modeling study (Pharris et al., 2019), it was noted that the CaM-binding domain on CaMKII overlaps significantly with the region on the CaMKII regulatory domain immediately downstream of T286, near where PP1 would bind to catalyze dephosphorylation of T286. Therefore, the authors suggested that bound CaM might block or partially inhibit reversal by PP1 of CaMKII autophosphorylation. The suggestion that bound CaM might interfere with binding of PP1 is supported by structural studies of binding of the PP1 catalytic subunit to substrates (Bollen et al., 2010) and to the targeting subunit spinophilin (Ragusa et al., 2010). PP1 interacts with both classes of proteins over a wide area of its surface. This property of PP1 suggests that bound CaM would sterically hinder binding of PP1 to CaMKII in the vicinity of phosphorylated T286, and thus inhibit dephosphorylation.

To measure the effect of CaM-trapping on the postulated inhibition of PP1 dephosphorylation by bound CaM, we modified the reaction rules for dephosphorylation to allow PP1 to bind to a pCaMKII subunit only when the cam site is free. This situation represents the most extreme case in which bound CaM completely blocks PP1 binding. Because a PP1 holoenzyme likely binds to pCaMKII via more than one docking site (Bollen et al., 2010), it is also possible that bound CaM lowers the affinity for PP1 by steric hindrance, but does not block dephosphorylation completely. We did not investigate this latter possibility in the present study.

**Figure 10:**
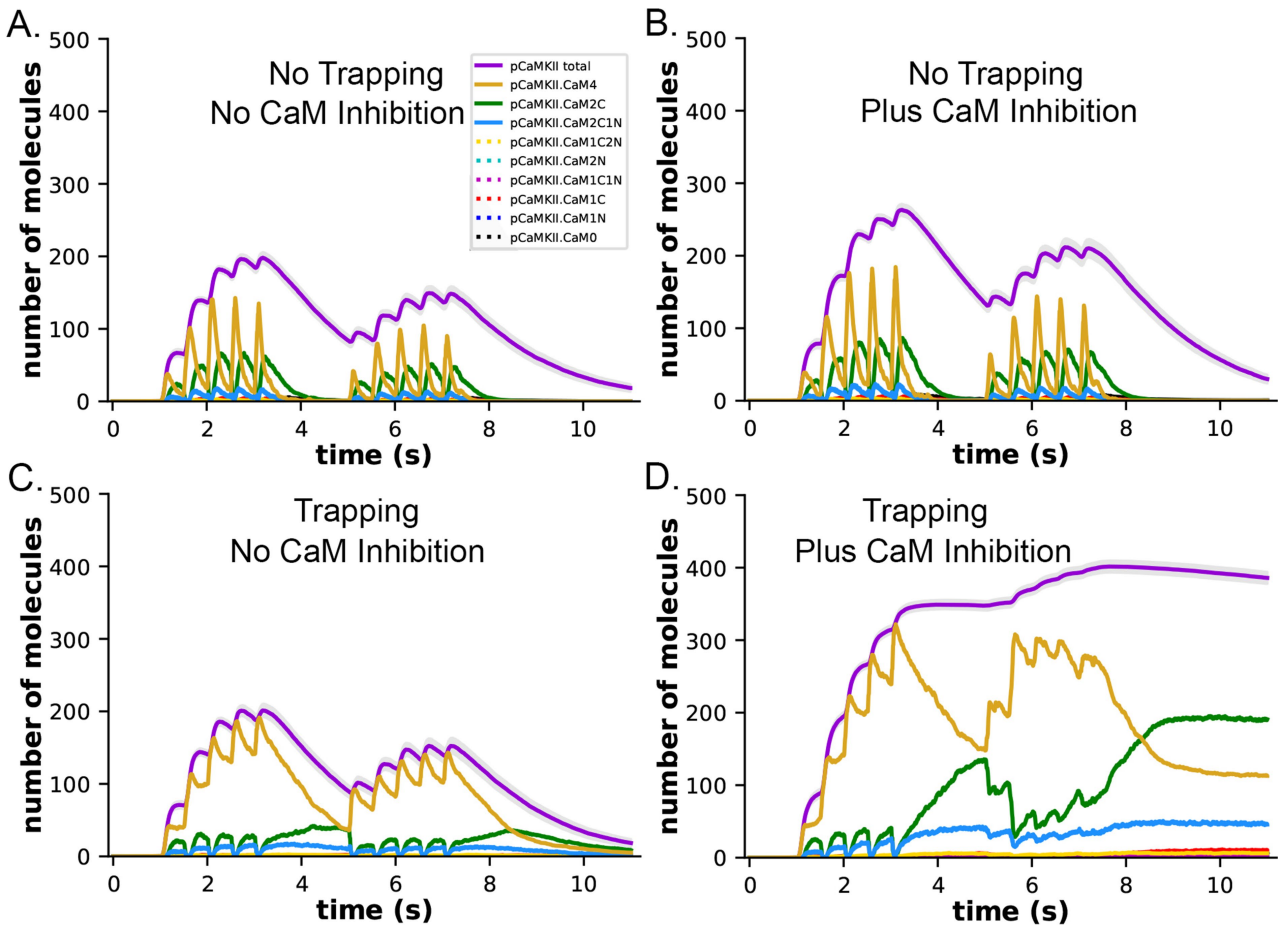
Dynamics of formation of pCaMKII by competition for binding between PP1 and CaM, in the presence and absence of CaM-trapping. **(A)** Data replotted from Figure 8A (no trapping) for comparison to **(B)**. **(B)** Dynamics of formation of pCaMKII in the presence of competition between CaM and PP1, and in the absence of trapping. **(C)** Data replotted from Figure 8B (trapping) for comparison to **(D)**. **(D)** Dynamics of formation of pCaMKII in the presence of competition between CaM and PP1, and in the presence of trapping. Each curve is the average of 50 simulations with 1.25 μM PP1, initiated with different random seeds.

**Figure 11:**
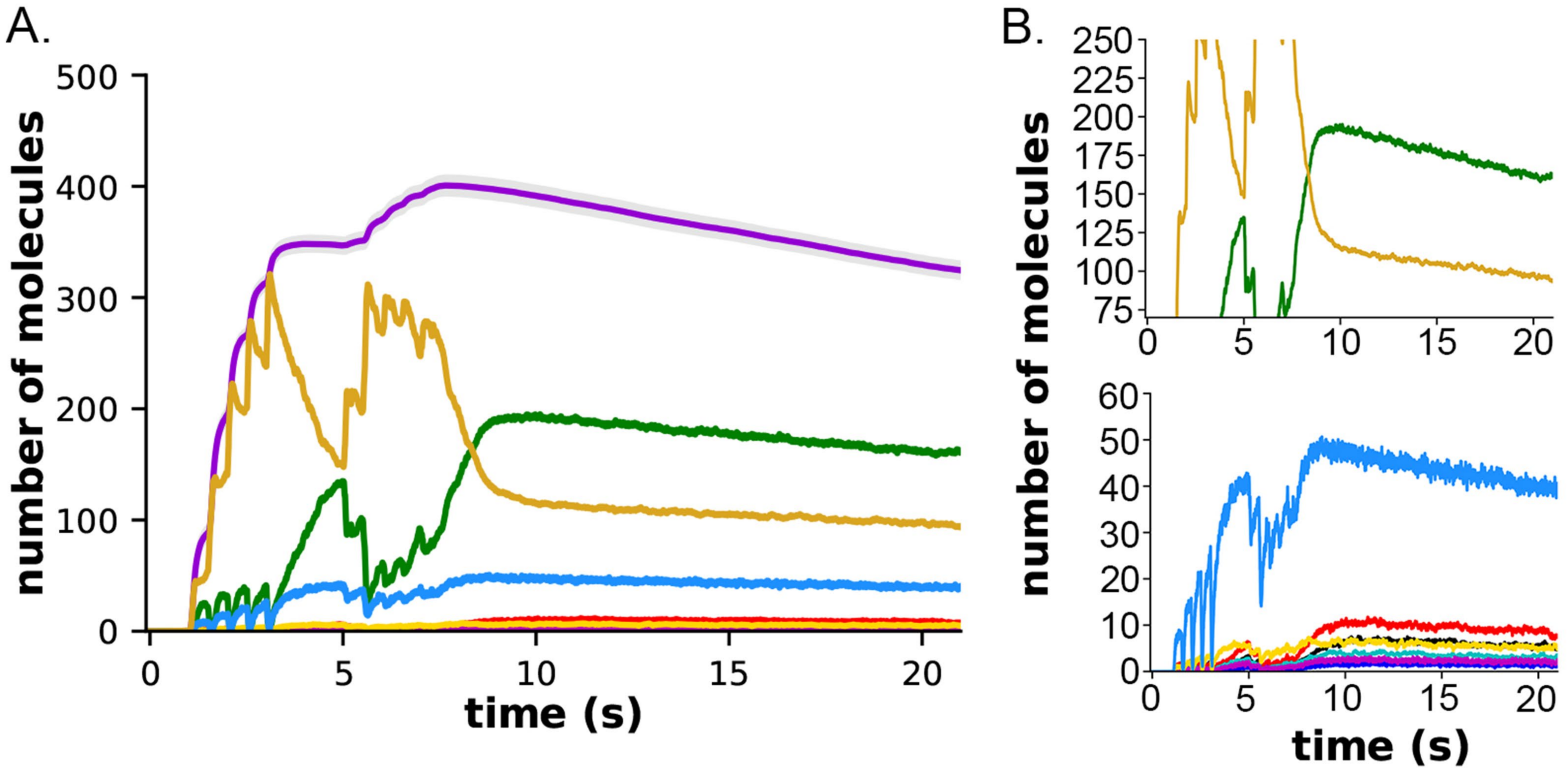
Formation and decay of pCaMKII and bound CaM species after the two-epoch stimulus. **(A)** The simulations depicted in Figure 10D were extended for an additional 10 s to measure the rate of dephosphorylation of pCaMKII in the presence of competition for binding between CaM and PP1. **(B)** Data from **(A)** plotted with magnified y-axes to show visible decay of bound CaM4, CaM2C (top) and CaM2C1N (bottom). The decay constant for dephosphorylation measured from this data are shown in Table 1.

In both the presence and absence of CaM-trapping, the inclusion of inhibition by CaM of dephosphorylation by PP1 increased the maximum formation of pCaMKII (Compare Figures 10A,C to B,D). The combined effect of inhibition by CaM and CaM-trapping was particularly profound, increasing the rate-constant 𝛕 for dephosphorylation from ~1.8 s to 60.4 s (Table 1) and resulting in autophosphorylation of approximately half of the CaMKII subunits at peak during the second epoch (Figures 10D, 11). Thus, the model predicts that the rate of decay of pCaMKII after a stimulus is intricately coupled to the relative affinities of binding among CaM, PP1, and CaMKII.

**Table 1:** Values of tau for dephosphorylation of pCaMKII in the non-trapping and trapping cases were determined at different simulated concentrations of PP1 from the data shown in Figures 7 and 10, 11, respectively, as described under Methods.

[PP1]	𝝉 Non-trapping		𝝉 Trapping		𝝉 Non-trapping CaM vs. PP1 competition		𝝉 Trapping CaM vs. PP1 competition	
	Epoch 1	Epoch 2	Epoch 1	Epoch 2	Epoch 1	Epoch 2	Epoch 1	Epoch 2
0.65 μM	5.0 s	4.9 s	5.01 s	4.8 s				
1.25 μM	1.9 s	1.7 s	2.0 s	1.8 s	2.3 s	1.9 s	60.4 s	60.4 s
2.5 μM	0.74 s	0.65 s	0.74 s	0.67 s				
5.0 μM	0.33 s	0.32 s	0.38 s	0.32 s				

The values of tau in the presence of competition for binding between CaM and PP1 were determined for the non-trapping case from the data shown in Figure 10B, and for the trapping case from Figure 11A. The tau for Epoch 1 was identical to that of Epoch 2 as determined by measuring the decay of pCaMKII from the peak at 4–5 s, which was identical to that of Epoch 2 from the peak at 7.7–8.7 s. Tau’s for CaM4, CaM2C, and CaM2C1N from Figure 11B were 61.7, 57.2, and 59.2, respectively.

**Figure 12:**
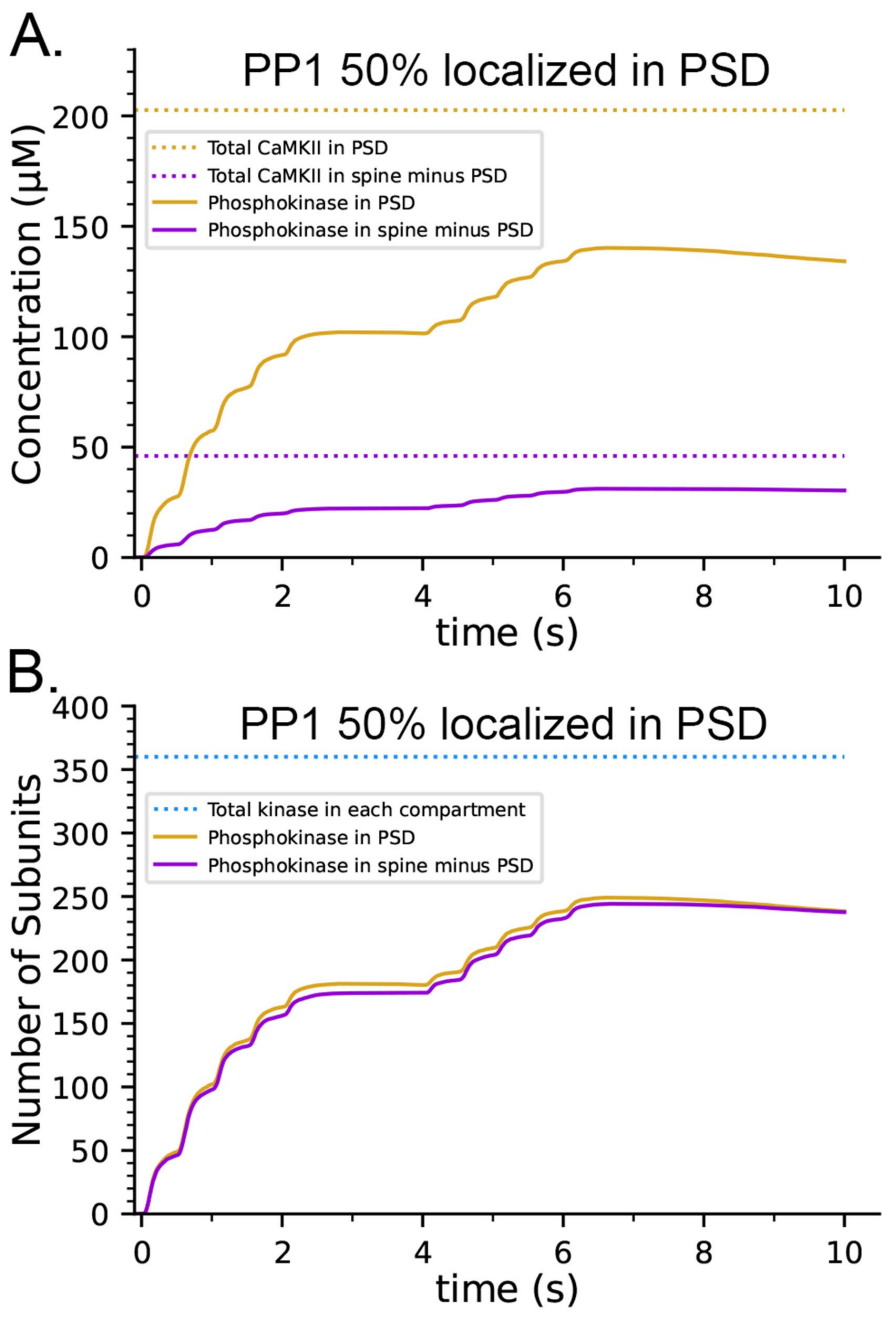
Effect of inhibition of PP1 activity by bound CaM on autophosphorylation when both CaMKII and PP1 are differentially localized in the PSD. **(A)** The concentration of pCaMKII reaches ~67% of the possible maximum of ~200 μm in the PSD. **(B)** The proportion of autophosphorylated subunits in a holoenzyme is ~67% in both compartments. Curves are averages of 50 simulations initiated with 50 different random seeds.

To evaluate the effect of inhibition by CaM when both CaMKII and PP1 are 50% localized in the PSD capsule, we added the inhibition rules to the localization model shown in Figure 9. In simulations with the modified model, the maximum concentration of pCaMKII formed in the PSD during the stimulus increased almost 3-fold from about ~50 μM (Figure 9B) to ~140 μM (Figure 12A). Although ~67% of subunits were autophosphorylated at the peak, the concentration of pCaMKII continued to decay slowly when the stimulus ended (Figures 12A,B). With 50% of CaMKII and PP1 both sequestered in the PSD capsule (Figure 12), the rate of decay (𝛕) in the capsule was 54.5 s and the rate in the cytosol outside the capsule was 72.0 s, compared to 60.4 s with neither sequestered (Table 1). These results mean that, under these physiological conditions, dephosphorylation by PP1 is not overwhelmed by the rate of recurring autophosphorylation within the holoenzyme, even when maximum autophosphorylation reaches 67% of the total subunits.

## Discussion

We developed a series of agent-based stochastic computational models to examine the predicted responses of CaMKII holoenzymes during the first 20 s after a two-epoch stimulus of medium strength in a realistic median-sized spine. The models are based upon four earlier, carefully-vetted published models (Bartol et al., 2015b; Pepke et al., 2010; Nadkarni et al., 2010; Ordyan et al., 2020). They contain physiologically realistic numbers of proteins situated in a spine from hippocampal neuropil (Bartol et al., 2015b). The numbers and kinetic parameters of the added CaMKII holoenzymes, CaM, and PP1 are drawn from measurements made over several years during biochemical and electrophysiological experiments cited in the text. The use of experimentally measured parameters and realistic numbers and spatial arrangements of the relevant proteins enabled testing of several hypotheses about the behavior of CaMKII under conditions present *in vivo*, that are difficult or impossible to test directly with experiments.

Previous experimental evidence has shown that PP1 is specifically enriched in synaptic spines (Strack et al., 1997; Ouimet et al., 1995), and regulates the direction and magnitude of changes in synaptic strength produced by different frequencies of stimulation (Foley et al., 2021). For example, the relationship between stimulus intensity and the direction of change in synaptic strength is altered by pharmacological agents that inhibit PP1 phosphatase activity (Brown et al., 2000; Coussens and Teyler, 1996). Under baseline conditions in slices from hippocampal area CA1, low frequency stimulation (1–3 Hz) leads to induction of long-term depression (LTD); stimulation at ~10 Hz is “neutral,” producing no change in synaptic strength, and higher frequency stimulation in the range of 25–100 Hz induces long-term potentiation (LTP) (Brown et al., 2000; Dudek and Bear, 1992). However, induction of LTD by stimulation at 1 Hz is blocked by application of inhibitors of PP1 (Mulkey et al., 1993). Conversely, induction of LTP is potentiated by inhibition of PP1 (Jouvanceau et al., 2006). Cyclic AMP-regulated inhibition of PP1 acts as a “gate” that determines whether LTP is produced by a given stimulus. Activation of the cAMP pathway by a variety of pharmacological agents permits induction of LTP by both widely spaced “HFS” stimulation (3 pulses of 900 APs at 100 Hz separated by 10 min, 107) and by “*θ* pulse” stimulation in the 5–12 Hz range (Brown et al., 2000). When the increase in cAMP is blocked, these stimuli do not induce LTP. Activation of β-adrenergic receptors by noradrenaline also increases LTP at hippocampal synapses through the cAMP pathway that inhibits PP1, suggesting that a similar mechanism underlies enhancement of learning by noradrenaline (Thomas et al., 1996). We chose the concentration of PP1, and its enzymatic parameters according to what we considered the best estimates of these parameters from the biochemical literature. Thus, the agreement between experimental findings showing sensitivity of synaptic plasticity to PP1 activity and the sensitivity of autophosphorylation to PP1 activity in our simulations (Figure 7), is an important first validation of the combined model.

CaM-trapping is a phenomenon discovered by Meyer et al. (1992) and elaborated on by Putkey and Waxham (1996). We decided to test the hypothesis that CaM-trapping causes an enhanced, non-linear increase in autophosphorylation of CaMKII during high frequency synaptic stimulation as suggested in earlier publications (Nicoll and Schulman, 2023; De Koninck and Schulman, 1998; Putney, 1998). To do this, simulations were performed in the presence and absence of the reaction rules that specify the change in affinity for CaM resulting from CaM-trapping. The simulations show that CaM-trapping does indeed prolong the association of CaM with autophosphorylated CaMKII subunits, but it does not alter the extent of autophosphorylation during the repeated stimuli in the 50 Hz frequency range that we used here. This is because the association of CaM with non-autophosphorylated subunits is not prolonged by trapping, and, thus, the probability of autophosphorylation is not increased during the second epoch.

We did, however, find that a function of CaM-trapping may be to dramatically alter the rate of reversal of autophosphorylation by dephosphorylation after a stimulus has ended. This finding is based on the structural prediction that binding of CaM to an autophosphorylated subunit of CaMKII is likely to sterically hinder binding of PP1 required for dephosphorylation of T286 (Pharris et al., 2019). The PP1 catalytic subunit interacts with substrates over an unusually large surface area comprising three different surface domains that surround the active site (Bollen et al., 2010). The recognition pockets for PP1 on substrates are usually more distant from the phosphorylated residue than the typical short consensus recognition sequences for protein kinases (Foley et al., 2021). Binding of PP1 to a substrate via two or more of its surface domains provides avidity that increases the K_M_ and thus, the rate of dephosphorylation. Conversely, reducing interaction of a substrate with one of the surface domains reduces the K_M_ and slows the rate of dephosphorylation (Bollen et al., 2010). In dendritic spines, the predominant targeting subunit for PP1 is spinophilin (Allen et al., 1997; Ouimet et al., 2004), which binds to spine actin filaments (Grossman et al., 2002). The spinophilin-PP1 holoenzyme, with a molecular mass of ~150 kDaL, is much larger than a CaMKII subunit. Bound spinophilin obstructs one of the three substrate-binding patches on the surface of the PP1 catalytic subunit (Ragusa et al., 2010). Thus, the spinophilin-PP1 holoenzyme would be expected to associate with substrates, including CaMKII, via the two remaining binding patches near the PP1 active site (Bollen et al., 2010). CaM binds to CaMKII at a site located ~7 residues downstream of T286 (Figure 2). Therefore, it is likely to partially, or fully, sterically hinder access of PP1 to phosphorylated T286.

**Figure 13:**
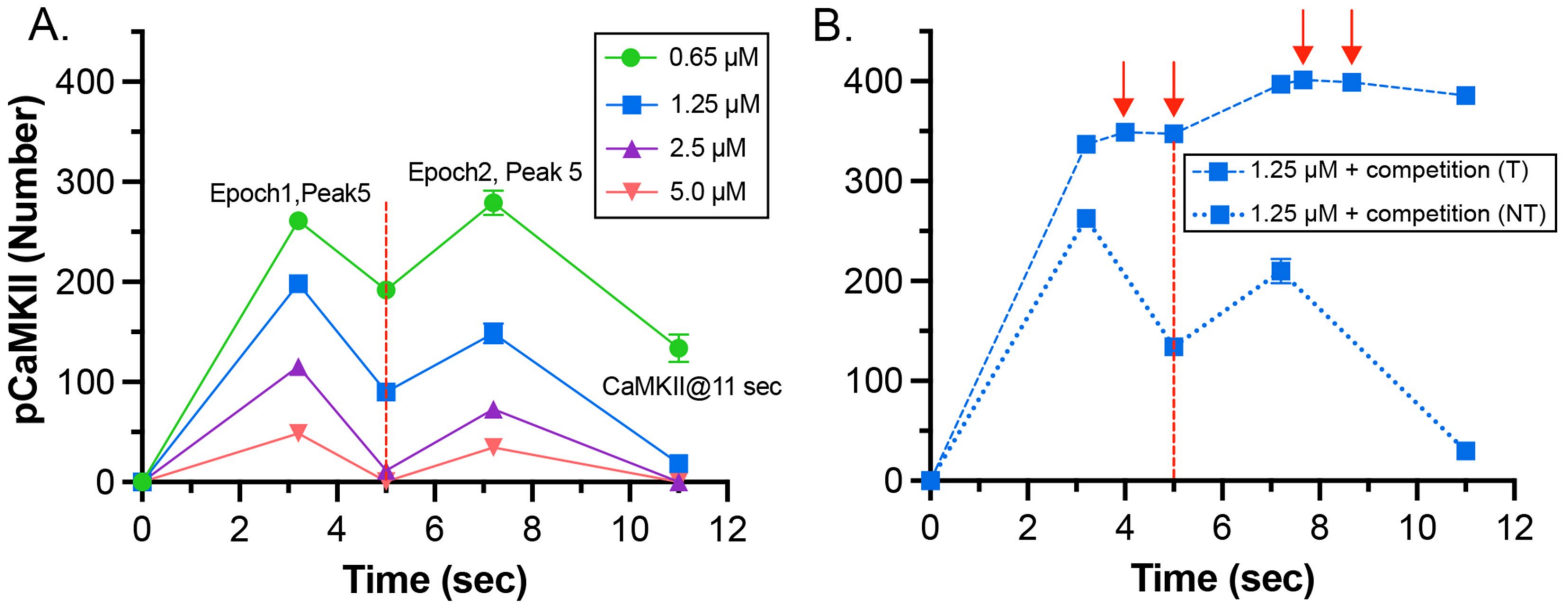
**(A)** Peak concentrations of pCaMKII during the two epoch stimulus in the presence of increasing concentrations of active PP1. Data are replotted for comparison from the results shown in Figure 7. The dotted red line at 5 s marks the time of initiation of the second epoch of the stimulus. Averages ± s.e.m. of 50 simulations. **(B)** Peak concentrations of pCaMKII during the two epoch stimulus including competition between binding of CaMx and PP1. Data with (T) and without CaM-trapping (NT) are replotted from the results shown in Figure 10D. In the graph of the model that includes CaM-trapping, points at 4 and 5 s and at 7.7 and 8.7 s are marked with red arrows. The differences in numbers of pCaMKII at these times were used to compare the decay time after the first and second epochs. S.e.m.’s for most of the measurements are smaller than the symbols.

To investigate the effect of competition between CaM-binding and dephosphorylation by PP1, we simulated the most extreme possibility which is complete block of dephosphorylation of T286 by bound CaM by adding a rule that PP1 cannot bind to pCaMKII when CaM is bound. In the absence of CaM-trapping, blocking of PP1 by bound CaM increased the rate of dephosphorylation (𝛕) by ~10–20% (Table 1). In contrast, when CaM-trapping was included in this model, dephosphorylation was dramatically slowed (Figure 13). The 𝛕 for dephosphorylation was increased 30-fold to 60.2 s, reflecting the much longer koff for trapped CaM. When bound CaM inhibited dephosphorylation of pCaMKII, the number of autophosphorylated subunits per CaMKII holoenzyme at the peak of the two-epoch 50 Hz stimulus doubled from ~200 pCaMKII subunits to ~400, out of the total of 720 subunits. Even this extreme competitive effect would prolong the lifetimes of autophosphorylated subunits for only a few minutes after the Ca^2+^ concentration returns to baseline. The slower dephosphorylation means that some pCaMKII subunits would remain phosphorylated for as long as 4 min (4𝛕). This finding is consistent with the experimental observation of Lee et al. (2009) that increased CaMKII phosphorylation is detectable in dendritic spines for a few minutes after two-photon glutamate uncaging to activate NMDA receptors. Thus, it also constitutes a second validation of our composite model against experimental results.

Yasuda et al. have used FRET methods to measure binding of CaM and transition of CaMKII to the activated state in single dendritic spines after activation of the spine by glutamate uncaging in the absence of Mg^2+^ (Chang et al., 2019; Chang et al., 2017). Although the Ca^2+^ stimulus is considerably stronger than our simulated stimulus, their measured decay times for activated wild type and mutant versions of CaMKII are similar to the times that our model predicts; and the structure of the coarse-grained model they propose to explain their results is compatible with our simulation results.

Competition between CaM-binding to pCaMKII and dephosphorylation of T286 by PP1 has not been adequately investigated experimentally. The actual effect of CaM-trapping *in vivo* on the rate of dephosphorylation of T286 by PP1 may be less than 100%. The only experimental study we are aware of that indirectly addressed the competition is Bradshaw et al. (2003). These authors sought to provide evidence for a bistable kinase switch comprised of CaMKII and PP1. Their experiments indicated that bound CaM did not interfere with dephosphorylation of Thr286 by PP1. However, the experiments were carried out at 0° C to suppress autophosphorylation of Thr305/306; in addition, they used a ratio of PP1 to CaMKII of 1/10, considerably higher than would be predicted *in vivo* from the measurements that we used in our model (~1/80). We propose that *in vitro* experiments with purified proteins to measure the rate of dephosphorylation of autophosphorylated CaMKII by PP1 in the presence and absence of Ca^2+^/CaM, would be an important test of the effect of CaM-trapping on the persistence of pCaMKII after a strong synaptic stimulus and would help to refine models of the biochemical response to strong synaptic stimulation. In addition, it will be important to determine the effect of autophosphorylation of thr305/306 on any inhibition (see Figure 1). It would be expected that autophosphorylation of thr305/306 following unbinding of Ca^2+^/CaM would prevent further binding of Ca^2+^/CaM and therefore decrease the length of time over which dephosphorylation is inhibited.

Many computational models of activation of CaMKII, incorporating various levels of detail and complexity, have been created previously. One conceptually attractive idea has been particularly persistent in these studies — the notion that CaMKII and PP1 together may form a bistable switch that shifts between an active highly autophosphorylated stable steady-state reflecting LTP and an inactive non-autophosphorylated stable steady-state reflecting LTD (Miller et al., 2005; Lisman et al., 2002; Zhabotinsky, 2000; Lisman and Zhabotinsky, 2001; Pi and Lisman, 2008; Michalski, 2013; Michalski, 2014). Because the notion of bistability focuses on steady state behavior, it has been encoded in deterministic equations that specify multiple steady states (Pi and Lisman, 2008). These models usually include a control parameter that can change the steady state from the low stable state to the high stable state and have been used in mathematical biology to hypothesize that this behavior can occur in complex biological systems (For an introduction, see Strogatz, 2018). As a result, the notion of bistability of CaMKII activity, while appealing in its simplicity, did not evolve from the measured biochemical interactions of CaMKII, CaM, protein phosphatases, or other proposed key proteins. The composite model that we present here is distinct from these earlier models which employed deterministic methods and did not attempt to deduce kinetic mechanisms involving small numbers of molecules and highly structured reaction spaces. Here we have examined the stochastic behavior of CaMKII that is dictated by our best experimental understanding of the numbers of proteins, rate constants of interaction, and subcellular structure in which CaMKII is activated in the first minutes of a complex synaptic stimulus (Bartol et al., 2015b; Pepke et al., 2010; Nadkarni et al., 2010; Ordyan et al., 2020). With our models, and other previous models that incorporate more biochemical detail (Pharris et al., 2019; Li et al., 2012), bistability of CaMKII modulated by PP1, as envisioned in Lisman and Zhabotinsky (2001), is not observed.

We show that the presence of CaM-trapping in combination with inhibition by CaM of PP1 binding to pCaMKII can lead to a prolonged, but not permanent, elevated level of pCaMKII following the end of a stimulus, perhaps lasting several minutes. This finding suggests that a form of kinetic regulation may be operating that is sometimes referred to as “kinetic proof-reading” (Hopfield, 1974; Huang et al., 2016). This mechanism involves a biochemical network that is structured in such a way that a series of reversible biochemical steps can lead to an essentially irreversible one. The irreversible step only occurs when the preceding reversible steps (for example, binding between two molecules) last long enough to trigger the next step. The canonical example of kinetic proof-reading is the selection of the correct amino acid-tRNA complex to match a codon on ribosomal bound mRNA during protein synthesis. Only an exact match between the tRNA and mRNA codons results in binding that lasts long enough for irreversible formation of the peptide bond to occur (Hopfield, 1974).

We propose that to induce LTP, autophosphorylation of CaMKII subunits does not need to reach a the high, irreversible steady-state postulated by the bistable switch theory (e.g., Lisman and Zhabotinsky, 2001; Lisman, 1994). Rather, the convergence of signals at the synapse must simply result in a transient high level of autophosphorylation that lasts long enough to trigger one or more downstream, essentially irreversible, steps causing potentiation of the synapse. One such ‘irreversible’ step might include binding of a critical number of active CaMKII subunits to the carboxyl tails of GluN2B subunits of NMDA-receptors (Chen et al., 2024; Bayer et al., 2001; Bayer et al., 2006). Another might be sufficient dissolution of the protein condensate between PSD-95 and synGAP to trigger irreversible remodeling of the PSD by addition of AMPARs and enlargement of the supporting actin cytoskeleton (Opazo and Choquet, 2011; Araki et al., 2015; Walkup et al., 2016; Opazo et al., 2010). Both of these events have been proposed to be critical for development and maintenance of LTP.

Several distinct signals can converge on a synapse to modulate the extent of autophosphorylation of CaMKII. The primary signal observed experimentally is repeated high frequency (50–100 Hz) synaptic activation that causes firing of the neuron (see Nicoll and Schulman, 2023; Sjostrom and Nelson, 2002). In addition, activation of the cAMP pathway can inhibit PP1, prolonging the lifetime of autophosphorylated CaMKII (Blitzer et al., 2005; Brown et al., 2000; Blitzer et al., 1998; Thomas et al., 1996; Blitzer et al., 1995). Src and Fyn protein kinases, activated by G-protein-coupled receptors, receptor tyrosine kinases, and/or cytokines increase the flux of Ca^2+^ through NMDARs, potentiating induction of LTP by high frequency stimulation (Salter and Kalia, 2004). Kinetic proof-reading would allow combinations of these signals that lead to sufficiently prolonged autophosphorylation of CaMKII in a synapse to induce LTP.

The version of kinetic proof-reading that we describe here is similar, but not identical, to the more abstract “cascade” type models proposed by theorists (Hayer and Bhalla, 2005; Fusi et al., 2005). It attempts to describe how convergence, within a few seconds or minutes, of different experimentally-measured, reversible biochemical signals at a synapse might trigger a structural change that would produce a relatively long-lasting increase in synaptic strength. It remains to be seen whether the kinetic mechanism we propose is related to the “cascades” proposed by theorists.

The model we have presented has limitations that can be explored in further studies. We have not included autophosphorylation of thr305/306 in the CaM-binding domain following activation of kinase subunits, and thus we have not explored the potential quantitative effect of this autophosphorylation on subsequent CaM-binding or on potential inhibition of dephosphorylation. We have not yet examined differences in the responses of heterogeneous holoenzymes (Chien et al., 2024), including mixed α and β holoenzymes. The β-subunits have a higher initial affinity for CaM, which may influence kinetics of activation (Miller and Kennedy, 1985). The parameters in the present model were measured or adjusted to a range of temperatures from ~25°C to ~34°C. An earlier study of parameter sensitivity suggested that autophosphorylation kinetics are relatively insensitive to small variations in binding rates of Ca^2+^ and calmodulin to CaMKII (Pepke et al., 2010); however, it will be important to test the quantitative effect of adjustment of all parameters to 37° C (assuming a Q_10_ of 2). The influence of CaM-binding proteins in addition to CaMKII that are known to be present at significant concentration in the spine can be explored in future simulations. These proteins include neurogranin (Ordyan et al., 2020; Gaertner et al., 2004) and synGAP (Walkup et al., 2016). Important aspects of the response of CaMKII to a stimulus occur over a slower time course than the initial intraholoenzyme autophosphorylation measured here. For example, interholoenzyme autophosphorylation may be significant at the high concentrations of holoenzyme within the spine (Lucic et al., 2023). Other downstream sequelae of activation that have been postulated to prolong Ca^2+^ independent activity include binding of active CaMKII to GluN2B (Tullis et al., 2023; Chen et al., 2024) and to cytoskeletal proteins (Walikonis et al., 2001; Robison et al., 2005). These mechanisms, as well as phosphorylation of synaptic targets of CaMKII (e.g., Walkup et al., 2016; Araki et al., 2015) can be explored making use of the “check-pointing” ability of MCell4, which facilitates prolonged simulation times.

Future investigations will include responses of CaMKII in spines of different sizes, the quantitative effect on autophosphorylation of more prolonged and/or lower and higher frequency stimuli. Importantly, we will investigate the effects of proteins that localize CaM and compete for binding of CaM, and of proteins that regulate PP1. MCell4 is designed to be a powerful tool for introduction of each of these elements in a stepwise fashion to permit dissection of the effects of each and ultimately an understanding of their combined effects.

## Data Availability

The datasets presented in this study can be found in online repositories. The names of the repository/repositories and accession number(s) can be found below: http://www.mcell.cnl.salk.edu/models/spatial-model-of-CaMKII-2024-1/; https://doi.org/10.5281/zenodo.12764450; http://mcell.org; http://github.com/mcellteam/mcell.

## Footnotes

^1^ https://senselab.med.yale.edu/ModelDB/ShowModel.cshtml?model=2488

^2^ matplotlib.org

^3^ www.graphpad.com

## References

[ref1] Allen P. B. Ouimet C. C. Greengard P. (1997). Spinophilin, a novel protein phosphatase1 binding protein localized to dendritic spines. Proc. Natl. Acad. Sci. U. S. A. 94, 9956–9961. doi: 10.1073/pnas.94.18.9956, PMID: 9275233 PMC23308

[ref2] Araki Y. Rajkovich K. E. Gerber E. E. Gamache T. R. Johnson R. C. Tran T. H. N.. (2024). SynGAP regulates synaptic plasticity and cognition independently of its catalytic activity. Science 383:eadk1291. doi: 10.1126/science.adk1291, PMID: 38422154 PMC11188940

[ref3] Araki Y. Zeng M. Zhang M. Huganir R. L. (2015). Rapid dispersion of SynGAP from synaptic spines triggers AMPA receptor insertion and spine enlargement during LTP. Neuron 85, 173–189. doi: 10.1016/j.neuron.2014.12.023, PMID: 25569349 PMC4428669

[ref4] Ascher P. Nowak L. (1988). The role of divalent cations in the*N*-methyl-D-aspartate responses of mouse central neurones in culture. J. Physiol. 399, 247–266. doi: 10.1113/jphysiol.1988.sp017078, PMID: 2457089 PMC1191662

[ref5] Bartol T. M. Bromer C. Kinney J. Chirillo M. A. Bourne J. N. Harris K. M.. (2015a). Nanoconnectomic upper bound on the variability of synaptic plasticity. Elife 4:e10778. doi: 10.7554/eLife.10778, PMID: 26618907 PMC4737657

[ref6] Bartol T. M. Keller D. X. Kinney J. P. Bajaj C. L. Harris K. M. Sejnowski T. J.. (2015b). Computational reconstitution of spine calcium transients from individual proteins. Front. Synaptic Neurosci. 7:17. doi: 10.3389/fnsyn.2015.00017, PMID: 26500546 PMC4595661

[ref7] Bayer K. U. De Koninck P. Leonard A. S. Hell J. W. Schulman H. (2001). Interaction with the NMDA receptor locks CaMKII in an active conformation. Nature 411, 801–805. doi: 10.1038/35081080, PMID: 11459059

[ref8] Bayer K. U. LeBel E. McDonald G. L. O'Leary H. Schulman H. De Koninck P. (2006). Transition from reversible to persistent binding of CaMKII to postsynaptic sites and NR2B. J. Neurosci. 26, 1164–1174. doi: 10.1523/JNEUROSCI.3116-05.2006, PMID: 16436603 PMC2890238

[ref9] Bayer K. U. Schulman H. (2019). CaM kinase: still inspiring at 40. Neuron 103, 380–394. doi: 10.1016/j.neuron.2019.05.033, PMID: 31394063 PMC6688632

[ref10] Bennett M. K. Erondu N. E. Kennedy M. B. (1983). Purification and characterization of a calmodulin-dependent protein kinase that is highly concentrated in brain. J. Biol. Chem. 258, 12735–12744. doi: 10.1016/S0021-9258(17)44239-6, PMID: 6313675

[ref11] Bhalla U. S. (2002). Biochemical signaling networks decode temporal patterns of synaptic input. J. Comput. Neurosci. 13, 49–62. doi: 10.1023/A:1019644427655, PMID: 12154335

[ref12] Bi G. Poo M.-M. (1998). Synaptic modifications in cultured hippocampal neurons: dependence on spike timing, synaptic strength, and postsynaptic cell type. J. Neurosci. 18, 10464–10472. doi: 10.1523/JNEUROSCI.18-24-10464.1998, PMID: 9852584 PMC6793365

[ref13] Biederer T. Kaeser P. S. Blanpied T. A. (2017). Transcellular nanoalignment of synaptic function. Neuron 96, 680–696. doi: 10.1016/j.neuron.2017.10.006, PMID: 29096080 PMC5777221

[ref14] Bliss T. V. P. Collingridge G. L. (1993). A synaptic model of memory: long-term potentiation in the hippocampus. Nature 361, 31–39. doi: 10.1038/361031a0, PMID: 8421494

[ref15] Blitzer R. D. Connor J. H. Brown G. P. Wong T. Shenolikar S. Iyengar R.. (1998). Gating of CaMKII by cAMP-regulated protein phosphatase activity during LTP. Science 280, 1940–1943. doi: 10.1126/science.280.5371.1940, PMID: 9632393

[ref16] Blitzer R. D. Iyengar R. Landau E. M. (2005). Postsynaptic signaling networks: cellular cogwheels underlying long-term plasticity. Biol. Psychiatry 57, 113–119. doi: 10.1016/j.biopsych.2004.02.031, PMID: 15652868

[ref17] Blitzer R. D. Wong T. Nouranifar R. Iyengar R. Landau E. M. (1995). Postsynaptic cAMP pathway gates early LTP in hippocampal CA1 region. Neuron 15, 1403–1414. doi: 10.1016/0896-6273(95)90018-7, PMID: 8845163

[ref18] Bollen M. Peti W. Ragusa M. J. Beullens M. (2010). The extended PP1 toolkit: designed to create specificity. Trends Biochem. Sci. 35, 450–458. doi: 10.1016/j.tibs.2010.03.002, PMID: 20399103 PMC3131691

[ref19] Bourne J. Harris K. M. (2007). Do thin spines learn to be mushroom spines that remember? Curr. Opin. Neurobiol. 17, 381–386. doi: 10.1016/j.conb.2007.04.009, PMID: 17498943

[ref20] Bradshaw J. M. Kubota Y. Meyer T. Schulman H. (2003). An ultrasensitive Ca2+/calmodulin-dependent protein kinase II-protein phosphatase 1 switch facilitates specificity in postsynaptic calcium signaling. Proc. Natl. Acad. Sci. U. S. A. 100, 10512–10517. doi: 10.1073/pnas.1932759100, PMID: 12928489 PMC193592

[ref21] Brown G. P. Blitzer R. D. Connor J. H. Wong T. Shenolikar S. Iyengar R.. (2000). Long-term potentiation induced by theta frequency stimulation is regulated by a protein phosphatase-1-operated gate. J. Neurosci. 20, 7880–7887. doi: 10.1523/JNEUROSCI.20-21-07880.2000, PMID: 11050107 PMC6772713

[ref22] Burgin K. E. Waxham M. N. Rickling S. Westgate S. A. Mobley W. C. Kelly P. T. (1990). In situ hybridization histochemistry of Ca^2+^calmodulin-dependent protein kinase in developing rat brain. J. Neurosci. 10, 1788–1798. doi: 10.1523/JNEUROSCI.10-06-01788.1990, PMID: 2162385 PMC6570308

[ref23] Carlisle H. J. Luong T. N. Medina-Marino A. Schenker L. T. Khorosheva E. M. Indersmitten T.. (2011). Deletion of densin-180 results in abnormal behaviors associated with mental illness and reduces mGluR5 and DISC1 in the postsynaptic density fraction. J. Neurosci. 31, 16194–16207. doi: 10.1523/JNEUROSCI.5877-10.2011, PMID: 22072671 PMC3235477

[ref24] Chang J. Y. Nakahata Y. Hayano Y. Yasuda R. (2019). Mechanisms of ca(2+)/calmodulin-dependent kinase II activation in single dendritic spines. Nat. Commun. 10:2784. doi: 10.1038/s41467-019-10694-z, PMID: 31239443 PMC6592955

[ref25] Chang J. Y. Parra-Bueno P. Laviv T. Szatmari E. M. Lee S. R. Yasuda R. (2017). CaMKII autophosphorylation is necessary for optimal integration of ca(2+) signals during LTP induction, but not maintenance. Neuron 94, 800–808.e4. doi: 10.1016/j.neuron.2017.04.041, PMID: 28521133 PMC5573161

[ref26] Chao L. H. Stratton M. M. Lee I. H. Rosenberg O. S. Levitz J. Mandell D. J.. (2011). A mechanism for tunable autoinhibition in the structure of a human Ca^2+^/calmodulin- dependent kinase II holoenzyme. Cell 146, 732–745. doi: 10.1016/j.cell.2011.07.038, PMID: 21884935 PMC3184253

[ref27] Chen X. Cai Q. Zhou J. Pleasure S. J. Schulman H. Zhang M.. (2024). CaMKII autophosphorylation is the only enzymatic event required for synaptic memory. Proc. Natl. Acad. Sci. U. S. A. 121:e2402783121. doi: 10.1073/pnas.2402783121, PMID: 38889145 PMC11214084

[ref28] Chien C. T. Puhl H. Vogel S. S. Molloy J. E. Chiu W. Khan S. (2024). Hub stability in the calcium calmodulin-dependent protein kinase II. Commun. Biol. 7:766. doi: 10.1038/s42003-024-06423-y, PMID: 38918547 PMC11199487

[ref29] Colbran R. J. Smith M. K. Schworer C. M. Fong Y. L. Soderling T. R. (1989). Regulatory domain of calcium/calmodulin-dependent protein kinase II. Mechanism of inhibition and regulation by phosphorylation. J. Biol. Chem. 264, 4800–4804, PMID: 2538462

[ref30] Colbran R. J. Soderling T. R. (1990). Calcium/calmodulin-independent autophosphorylation sites of calcium/calmodulin-dependent protein kinase II. Studies on the effect of phosphorylation of threonine 305/306 and serine 314 on calmodulin binding using synthetic peptides. J. Biol. Chem. 265, 11213–11219. doi: 10.1016/S0021-9258(19)38578-3, PMID: 2162839

[ref31] Coussens C. Teyler T. J. (1996). Protein kinase and phosphatase activity regulate the form of synaptic plasticity expressed. Synapse 24, 97–103. doi: 10.1002/(SICI)1098-2396(199610)24:2<97::AID-SYN1>3.0.CO;2-9, PMID: 8890451

[ref32] De Koninck P. Schulman H. (1998). Sensitivity of CaM kinase II to the frequency of Ca^2+^oscillations. Science 279, 227–230. doi: 10.1126/science.279.5348.227, PMID: 9422695

[ref33] Diering G. H. Huganir R. L. (2018). The AMPA receptor code of synaptic plasticity. Neuron 100, 314–329. doi: 10.1016/j.neuron.2018.10.018, PMID: 30359599 PMC6214363

[ref34] Dobrunz L. E. Huang E. P. Stevens C. F. (1997). Very short-term plasticity in hippocampal synapses. Proc. Natl. Acad. Sci. U. S. A. 94, 14843–14847. doi: 10.1073/pnas.94.26.14843, PMID: 9405701 PMC25125

[ref35] Dudek S. M. Bear M. F. (1992). Homosynaptic long-term depression in area CA1 of hippocampus and the effects of NMDA receptor blockade. Proc. Natl. Acad. Sci. U. S. A. 89, 4363–4367. doi: 10.1073/pnas.89.10.4363, PMID: 1350090 PMC49082

[ref36] Erondu N. E. Kennedy M. B. (1985). Regional distribution of type II Ca^2+^/calmodulin-dependent protein kinase in rat brain. J. Neurosci. 5, 3270–3277. doi: 10.1523/JNEUROSCI.05-12-03270.1985, PMID: 4078628 PMC6565219

[ref37] Faeder J. R. Blinov M. L. Hlavacek W. S. (2009). Rule-based modeling of biochemical systems with BioNetGen. Methods Mol. Biol. 500, 113–167. doi: 10.1007/978-1-59745-525-1_5, PMID: 19399430

[ref38] Foley K. McKee C. Nairn A. C. Xia H. (2021). Regulation of synaptic transmission and plasticity by protein phosphatase 1. J. Neurosci. 41, 3040–3050. doi: 10.1523/JNEUROSCI.2026-20.2021 33827970 PMC8026358

[ref39] Fusi S. Drew P. J. Abbott L. F. (2005). Cascade models of synaptically stored memories. Neuron 45, 599–611. doi: 10.1016/j.neuron.2005.02.001, PMID: 15721245

[ref40] Gaertner T. R. Kolodziej S. J. Wang D. Kobayashi R. Koomen J. M. Stoops J. K.. (2004). Comparative analyses of the 3-dimensional structures and enzymatic properties of the alpha, beta, gamma and delta isoforms of Ca2+−calmodulin dependent protein kinase II. J. Biol. Chem. 279, 12484–12494. doi: 10.1074/jbc.M313597200, PMID: 14722083

[ref41] Gaertner T. R. Putkey J. A. Waxham M. N. (2004). RC3/Neurogranin and Ca2+/calmodulin-dependent protein kinase II produce opposing effects on the affinity of calmodulin for calcium. J. Biol. Chem. 279, 39374–39382. doi: 10.1074/jbc.M405352200, PMID: 15262982

[ref42] Giese K. P. Fedorov N. B. Filipkowski R. K. Silva A. J. (1998). Autophosphorylation at Thr286 of the alpha calcium-calmodulin kinase II in LTP and learning. Science 279, 870–873. doi: 10.1126/science.279.5352.870, PMID: 9452388

[ref43] Gillespie D. T. (1977). Exact stochastic simulation of coupled chemical reactions. J. Phys. Chem. 81, 2340–2361. doi: 10.1021/j100540a008

[ref44] Gillespie D. T. (2007). Stochastic simulation of chemical kinetics. Annu. Rev. Phys. Chem. 58, 35–55. doi: 10.1146/annurev.physchem.58.032806.104637, PMID: 17037977

[ref45] Goda Y. Stevens C. F. (1994). Two components of transmitter release at a central synapse. Proc. Natl. Acad. Sci. U. S. A. 91, 12942–12946. doi: 10.1073/pnas.91.26.12942, PMID: 7809151 PMC45556

[ref46] Goncalves J. Bartol T. M. Camus C. Levet F. Menegolla A. P. Sejnowski T. J.. (2020). Nanoscale co-organization and coactivation of AMPAR, NMDAR, and mGluR at excitatory synapses. Proc. Natl. Acad. Sci. U. S. A. 117, 14503–14511. doi: 10.1073/pnas.1922563117, PMID: 32513712 PMC7321977

[ref47] Grossman S. D. Hsieh-Wilson L. C. Allen P. B. Nairn A. C. Greengard P. (2002). The actin-binding domain of spinophilin is necessary and sufficient for targeting to dendritic spines. NeuroMolecular Med. 2, 61–70. doi: 10.1385/NMM:2:1:61, PMID: 12230305

[ref48] Gupta S. Czech J. Kuczewski R. Bartol T. M. Sejnowski T. J. Lee R. E. C.. (2018). Spatial stochastic modeling with MCell and CellBlender. arXiv [Preprint]. doi: 10.48550/arXiv.1810.00499

[ref49] Haas K. T. Compans B. Letellier M. Bartol T. M. Grillo-Bosch D. Sejnowski T. J.. (2018). Pre-post synaptic alignment through neuroligin-1 tunes synaptic transmission efficiency. Elife 7:7. doi: 10.7554/eLife.31755, PMID: 30044218 PMC6070337

[ref50] Hanson P. I. Meyer T. Stryer L. Schulman H. (1994). Dual role of calmodulin in autophosphorylation of multifunctional CaM kinase may underlie decoding of calcium signals. Neuron 12, 943–956. doi: 10.1016/0896-6273(94)90306-9, PMID: 8185953

[ref51] Harnett M. T. Makara J. K. Spruston N. Kath W. L. Magee J. C. (2012). Synaptic amplification by dendritic spines enhances input cooperativity. Nature 491, 599–602. doi: 10.1038/nature11554, PMID: 23103868 PMC3504647

[ref52] Harris K. M. Weinberg R. J. (2012). Ultrastructure of synapses in the mammalian brain. Cold Spring Harb. Perspect. Biol. 4:a005587. doi: 10.1101/cshperspect.a005587 22357909 PMC3331701

[ref53] Hayer A. Bhalla U. S. (2005). Molecular switches at the synapse emerge from receptor and kinase traffic. PLoS Comput. Biol. 1, 137–154. doi: 10.1371/journal.pcbi.0010020 16110334 PMC1185646

[ref54] Hell J. W. (2014). CaMKII: claiming center stage in postsynaptic function and organization. Neuron 81, 249–265. doi: 10.1016/j.neuron.2013.12.024, PMID: 24462093 PMC4570830

[ref55] Holmes W. R. (2000). Models of calmodulin trapping and CaM kinase II activation in a dendritic spine. J. Comput. Neurosci. 8, 65–86. doi: 10.1023/A:1008969032563 10798500

[ref56] Hopfield J. J. (1974). Kinetic proofreading: a new mechanism for reducing errors in biosynthetic processes requiring high specificity. Proc. Natl. Acad. Sci. U. S. A. 71, 4135–4139. doi: 10.1073/pnas.71.10.4135, PMID: 4530290 PMC434344

[ref57] Huang W. Y. Yan Q. Lin W. C. Chung J. K. Hansen S. D. Christensen S. M.. (2016). Phosphotyrosine-mediated LAT assembly on membranes drives kinetic bifurcation in recruitment dynamics of the Ras activator SOS. Proc. Natl. Acad. Sci. USA 113, 8218–8223. doi: 10.1073/pnas.1602602113, PMID: 27370798 PMC4961118

[ref58] Husar A. Ordyan M. Garcia G. C. Yancey J. G. Saglam A. S. Faeder J. R.. (2024). MCell4 with BioNetGen: a Monte Carlo simulator of rule-based reaction-diffusion systems with Python interface. PLoS Comput. Biol. 20:e1011800. doi: 10.1371/journal.pcbi.1011800, PMID: 38656994 PMC11073787

[ref59] Ingebritsen T. S. Stewart A. A. Cohen P. (1983). The protein phosphatases involved in cellular regulation. 6. Measurement of type-1 and type-2 protein phosphatases in extracts of mammalian tissues; an assessment of their physiological roles. Eur. J. Biochem. 132, 297–307. doi: 10.1111/j.1432-1033.1983.tb07362.x, PMID: 6301829

[ref60] Jedrzejewska-Szmek J. Dorman D. B. Blackwell K. T. (2023). Making time and space for calcium control of neuron activity. Curr. Opin. Neurobiol. 83:102804. doi: 10.1016/j.conb.2023.102804, PMID: 37913687 PMC10842147

[ref61] Jouvanceau A. Hedou G. Potier B. Kollen M. Dutar P. Mansuy I. M. (2006). Partial ihibition of PP1 alters bidirectional synaptic plasticity in the hippocampus. Eur. J. Neurosci. 24, 464–572. doi: 10.1111/j.1460-9568.2006.04938 16903858

[ref62] Kakiuchi S. Yasuda S. Yamazaki R. Teshima Y. Kanda K. Kakiuchi R.. (1982). Quantitative determinations of calmodulin in the supernatant and particulate fractions of mammalian tissues. J. Biochem. 92, 1041–1048. doi: 10.1093/oxfordjournals.jbchem.a134019, PMID: 7174634

[ref63] Keller D. X. Franks K. M. Bartol T. M. Jr. Sejnowski T. J. (2008). Calmodulin activation by calcium transients in the postsynaptic density of dendritic spines. PLoS One 3:e2045. doi: 10.1371/journal.pone.0002045, PMID: 18446197 PMC2312328

[ref64] Kennedy M. B. (2000). Signal-processing machines at the postsynaptic density. Science 290, 750–754. doi: 10.1126/science.290.5492.750, PMID: 11052931

[ref65] Kennedy M. B. (2013). Synaptic signaling in learning and memory. Cold Spring Harb. Perspect. Biol. 8:a016824. doi: 10.1101/cshperspect.a016824, PMID: 24379319 PMC4743082

[ref66] Kennedy M. B. Beale H. C. Carlisle H. J. Washburn L. R. (2005). Integration of biochemical signalling in spines. Nat. Rev. Neurosci. 6, 423–434. doi: 10.1038/nrn1685, PMID: 15928715

[ref67] Kennedy M. B. Bennett M. K. Erondu N. E. (1983). Biochemical and immunochemical evidence that the "major postsynaptic density protein" is a subunit of a calmodulin-dependent protein kinase. Proc. Natl. Acad. Sci. U. S. A. 80, 7357–7361. doi: 10.1073/pnas.80.23.7357, PMID: 6580651 PMC390054

[ref68] Kerr R. A. Bartol T. M. Kaminsky B. Dittrich M. Chang J. C. Baden S. B.. (2008). Fast Monte Carlo simulation methods for biological reaction-diffusion Systems in Solution and on surfaces. SIAM J. Sci. Comput. 30, 3126–3149. doi: 10.1137/070692017, PMID: 20151023 PMC2819163

[ref69] Lee S. J. Escobedo-Lozoya Y. Szatmari E. M. Yasuda R. (2009). Activation of CaMKII in single dendritic spines during long-term potentiation. Nature 458, 299–304. doi: 10.1038/nature07842, PMID: 19295602 PMC2719773

[ref70] Li S. Raychaudhuri S. Lee S. A. Brockmann M. M. Wang J. Kusick G.. (2021). Asynchronous release sites align with NMDA receptors in mouse hippocampal synapses. Nat. Commun. 12:677. doi: 10.1038/s41467-021-21004-x, PMID: 33514725 PMC7846561

[ref71] Li L. Stefan M. I. Le Novere N. (2012). Calcium input frequency, duration and amplitude differentially modulate the relative activation of calcineurin and CaMKII. PLoS One 7:e43810. doi: 10.1371/journal.pone.0043810, PMID: 22962589 PMC3433481

[ref72] Lisman J. E. (1985). A mechanism for memory storage insensitive to molecular turnover: a bistable autophosphorylating kinase. Proc. Natl. Acad. Sci. U. S. A. 82, 3055–3057. doi: 10.1073/pnas.82.9.3055, PMID: 2986148 PMC397705

[ref73] Lisman J. (1994). The CaM kinase II hypothesis for the storage of synaptic memory. Trends Neurosci. 17, 406–412. doi: 10.1016/0166-2236(94)90014-0, PMID: 7530878

[ref74] Lisman J. Schulman H. Cline H. (2002). The molecular basis of CaMKII function in synaptic and behavioural memory. Nat. Rev. Neurosci. 3, 175–190. doi: 10.1038/nrn753, PMID: 11994750

[ref75] Lisman J. E. Zhabotinsky A. M. (2001). A model of synaptic memory: a CaMKII/PP1 switch that potentiates transmission by organizing an AMPA receptor anchoring assembly. Neuron 31, 191–201. doi: 10.1016/S0896-6273(01)00364-6, PMID: 11502252

[ref76] Lucic I. Heluin L. Jiang P. L. Castro Scalise A. G. Wang C. Franz A.. (2023). CaMKII autophosphorylation can occur between holoenzymes without subunit exchange. Elife 12:12. doi: 10.7554/eLife.86090, PMID: 37566455 PMC10468207

[ref77] Magee J. C. Johnston D. (1997). A synaptically controlled, associative signal for Hebbian plasticity in hippocampal neurons. Science 275, 209–213. doi: 10.1126/science.275.5297.209, PMID: 8985013

[ref78] Mainen Z. F. Joerges J. Huguenard J. R. Sejnowski T. J. (1995). A model of spike initiation in neocortical pyramidal neurons. Neuron 15, 1427–1439. doi: 10.1016/0896-6273(95)90020-9, PMID: 8845165

[ref79] Malenka R. C. Bear M. F. (2004). LTP and LTD: an embarrassment of riches. Neuron 44, 5–21. doi: 10.1016/j.neuron.2004.09.012, PMID: 15450156

[ref80] Markram H. Lübke J. Frotscher M. Sakmann B. (1997). Regulation of synaptic efficacy by coincidence of postsynaptic APs and EPSPs. Science 275, 213–215. doi: 10.1126/science.275.5297.213, PMID: 8985014

[ref81] McAvoy T. Allen P. B. Obaishi H. Nakanishi H. Takai Y. Greengard P.. (1999). Regulation of neurabin I interaction with protein phosphatase 1 by phosphorylation. Biochemistry 38, 12943–12949. doi: 10.1021/bi991227d, PMID: 10504266

[ref82] Meyer T. Hanson P. I. Stryer L. Schulman H. (1992). Calmodulin trapping by calcium-calmodulin dependent protein kinase. Science 256, 1199–1202. doi: 10.1126/science.256.5060.1199, PMID: 1317063

[ref83] Michalski P. J. (2013). The delicate bistability of CaMKII. Biophys. J. 105, 794–806. doi: 10.1016/j.bpj.2013.06.038, PMID: 23931327 PMC3736660

[ref84] Michalski P. J. (2014). First demonstration of bistability in CaMKII, a memory-related kinase. Biophys. J. 106, 1233–1235. doi: 10.1016/j.bpj.2014.01.037, PMID: 24655498 PMC3984987

[ref85] Miller S. G. Kennedy M. B. (1985). Distinct forebrain and cerebellar isozymes of type II Ca^2+^/calmodulin-dependent protein kinase associate differently with the postsynaptic density fraction. J. Biol. Chem. 260, 9039–9046. doi: 10.1016/S0021-9258(17)39454-1, PMID: 4019461

[ref86] Miller S. G. Kennedy M. B. (1986). Regulation of brain type II Ca^2+^/calmodulin-dependent protein kinase by autophosphorylation: a Ca^2+^-triggered molecular switch. Cell 44, 861–870. doi: 10.1016/0092-8674(86)90008-5, PMID: 3006921

[ref87] Miller S. G. Patton B. L. Kennedy M. B. (1988). Sequences of autophosphorylation sites in neuronal type II CaM kinase that control Ca^2+^-independent activity. Neuron 1, 593–604. doi: 10.1016/0896-6273(88)90109-2, PMID: 2856100

[ref88] Miller P. Zhabotinsky A. M. Lisman J. E. Wang X. J. (2005). The stability of a stochastic CaMKII switch: dependence on the number of enzyme molecules and protein turnover. PLoS Biol. 3:e107. doi: 10.1371/journal.pbio.0030107, PMID: 15819604 PMC1069645

[ref89] Monroe J. D. Heathcote R. D. (2013). Protein phosphatases regulate the growth of developing neurites. Int. J. Dev. Neurosci. 31, 250–257. doi: 10.1016/j.ijdevneu.2013.01.005, PMID: 23376726

[ref90] Mulkey R. M. Herron C. E. Malenka R. C. (1993). An essential role for protein phosphatases in hippocampal long-term depression. Science 261, 1051–1055. doi: 10.1126/science.8394601, PMID: 8394601

[ref91] Mullins C. Fishell G. Tsien R. W. (2016). Unifying views of autism Spectrum disorders: a consideration of autoregulatory feedback loops. Neuron 89, 1131–1156. doi: 10.1016/j.neuron.2016.02.017, PMID: 26985722 PMC5757244

[ref92] Nadkarni S. Bartol T. M. Sejnowski T. J. Levine H. (2010). Modelling vesicular release at hippocampal synapses. PLoS Comput. Biol. 6:e1000983. doi: 10.1371/journal.pcbi.1000983, PMID: 21085682 PMC2978677

[ref93] Nadkarni S. Bartol T. M. Stevens C. F. Sejnowski T. J. Levine H. (2012). Short-term plasticity constrains spatial organization of a hippocampal presynaptic terminal. Proc. Natl. Acad. Sci. U. S. A. 109, 14657–14662. doi: 10.1073/pnas.1211971109, PMID: 22908295 PMC3437845

[ref94] Nicoll R. A. Schulman H. (2023). Synaptic memory and CaMKII. Physiol. Rev. 103, 2877–2925. doi: 10.1152/physrev.00034.2022, PMID: 37290118 PMC10642921

[ref95] Oh J. S. Manzerra P. Kennedy M. B. (2004). Regulation of the neuron-specific Ras GTPase-activating protein, synGAP, by Ca2+/calmodulin-dependent protein kinase II. J. Biol. Chem. 279, 17980–17988. doi: 10.1074/jbc.M314109200, PMID: 14970204

[ref96] Opazo P. Choquet D. (2011). A three-step model for the synaptic recruitment of AMPA receptors. Mol. Cell. Neurosci. 46, 1–8. doi: 10.1016/j.mcn.2010.08.014, PMID: 20817097

[ref97] Opazo P. Labrecque S. Tigaret C. M. Frouin A. Wiseman P. W. De Koninck P.. (2010). CaMKII triggers the diffusional trapping of surface AMPARs through phosphorylation of stargazin. Neuron 67, 239–252. doi: 10.1016/j.neuron.2010.06.007, PMID: 20670832

[ref98] Ordyan M. Bartol T. Kennedy M. Rangamani P. Sejnowski T. (2020). Interactions between calmodulin and neurogranin govern the dynamics of CaMKII as a leaky integrator. PLoS Comput. Biol. 16:e1008015. doi: 10.1371/journal.pcbi.1008015, PMID: 32678848 PMC7390456

[ref99] Ouimet C. C. Da Cruz E Silva E. F. Greengard P. (1995). The alpha and gamma 1 isoforms of protein phosphatase 1 are highly and specifically concentrated in dendritic spines. Proc. Natl. Acad. Sci. U. S. A. 92, 3396–3400. doi: 10.1073/pnas.92.8.3396, PMID: 7724573 PMC42173

[ref100] Ouimet C. C. Katona I. Allen P. Freund T. F. Greengard P. (2004). Cellular and subcellular distribution of spinophilin, a PP1 regulatory protein that bundles F-actin in dendritic spines. J. Comp. Neurol. 479, 374–388. doi: 10.1002/cne.20313, PMID: 15514983

[ref101] Patton B. L. Miller S. G. Kennedy M. B. (1990). Activation of type II calcium/calmodulin-dependent protein kinase by Ca^2+^/calmodulin is inhibited by autophosphorylation of threonine within the calmodulin-binding domain. J. Biol. Chem. 265, 11204–11212. doi: 10.1016/S0021-9258(19)38577-1, PMID: 2162838

[ref102] Pepke S. Kinzer-Ursem T. Mihalas S. Kennedy M. B. (2010). A dynamic model of interactions of Ca^2+^, calmodulin, and catalytic subunits of Ca^2+^/calmodulin-dependent protein kinase II. PLoS Comput. Biol. 6:e1000675. doi: 10.1371/journal.pcbi.1000675, PMID: 20168991 PMC2820514

[ref103] Pharris M. C. Patel N. M. VanDyk T. G. Bartol T. M. Sejnowski T. J. Kennedy M. B.. (2019). A multi-state model of the CaMKII dodecamer suggests a role for calmodulin in maintenance of autophosphorylation. PLoS Comput. Biol. 15:e1006941. doi: 10.1371/journal.pcbi.1006941, PMID: 31869343 PMC6957207

[ref104] Pi H. J. Lisman J. E. (2008). Coupled phosphatase and kinase switches produce the tristability required for long-term potentiation and long-term depression. J. Neurosci. 28, 13132–13138. doi: 10.1523/JNEUROSCI.2348-08.2008, PMID: 19052204 PMC2620235

[ref105] Platholi J. Hemmings H. C. Jr. (2021). Modulation of dendritic spines by protein phosphatase-1. Adv. Pharmacol. 90, 117–144. doi: 10.1016/bs.apha.2020.10.001, PMID: 33706930 PMC8973313

[ref106] Putkey J. A. Waxham M. N. (1996). A peptide model for calmodulin trapping by calcium/calmodulin-dependent protein kinase II. J. Biol. Chem. 271, 29619–29623. doi: 10.1074/jbc.271.47.29619, PMID: 8939892

[ref107] Putney J. W. Jr. (1998). Calcium signaling: up, down, up, down…what's the point? Science 279, 191–192. doi: 10.1126/science.279.5348.191, PMID: 9446226

[ref108] Racca C. Stephenson F. A. Streit P. Roberts J. D. Somogyi P. (2000). NMDA receptor content of synapses in stratum radiatum of the hippocampal CA1 area. J. Neurosci. 20, 2512–2522. doi: 10.1523/JNEUROSCI.20-07-02512.2000, PMID: 10729331 PMC6772245

[ref109] Ragusa M. J. Dancheck B. Critton D. A. Nairn A. C. Page R. Peti W. (2010). Spinophilin directs protein phosphatase 1 specificity by blocking substrate binding sites. Nat. Struct. Mol. Biol. 17, 459–464. doi: 10.1038/nsmb.1786 20305656 PMC2924587

[ref110] Rammes G. (2023). Molecular mechanism of Alzheimer’s disease. Int. J. Mol. Sci. 24:16837. doi: 10.3390/ijms242316837, PMID: 38069160 PMC10706155

[ref111] Rellos P. Pike A. C. W. Niesen F. H. Salah E. Lee W. H. von Delft F.. (2010). Structure of the CaMKIIdelta/calmodulin complex reveals the molecular mechanism of CaMKII kinase activation. PLoS Biol. 8:e1000426. doi: 10.1371/journal.pbio.1000426, PMID: 20668654 PMC2910593

[ref112] Robison A. J. Bartlett R. K. Bass M. A. Colbran R. J. (2005). Differential modulation of Ca2+/calmodulin-dependent protein kinase II activity by regulated interactions with N-methyl-D-aspartate receptor NR2B subunits and alpha-actinin. J. Biol. Chem. 280, 39316–39323. doi: 10.1074/jbc.M508189200, PMID: 16172120

[ref113] Rosenberg O. S. Deindl S. Sung R. J. Nairn A. C. Kuriyan J. (2005). Structure of the autoinhibited kinase domain of CaMKII and SAXS analysis of the holoenzyme. Cell 123, 849–860. doi: 10.1016/j.cell.2005.10.029, PMID: 16325579

[ref114] Sabatini B. L. Oertner T. G. Svoboda K. (2002). The life cycle of Ca^2+^ions in dendritic spines. Neuron 33, 439–452. doi: 10.1016/S0896-6273(02)00573-1, PMID: 11832230

[ref115] Salter M. W. Kalia L. V. (2004). Src kinases: a hub for NMDA receptor regulation. Nat. Rev. Neurosci. 5, 317–328. doi: 10.1038/nrn1368, PMID: 15034556

[ref116] Sandal P. Jong C. J. Merrill R. A. Song J. Strack S. (2021). Protein phosphatase 2A - structure, function and role in neurodevelopmental disorders. J. Cell Sci. 134:jcs248187. doi: 10.1242/jcs.248187, PMID: 34228795 PMC8277144

[ref117] Schworer C. M. Colbran R. J. Keefer J. R. Soderling T. R. (1988). Ca2+/calmodulin-dependent protein kinase II. Identification of a regulatory autophosphorylation site adjacent to the inhibitory and calmodulin-binding domains. J. Biol. Chem. 263, 13486–13489. doi: 10.1016/S0021-9258(18)68264-X, PMID: 3417668

[ref118] Scolnick E. M. (2017). The path to new therapies for schizophrenia and bipolar illness. FASEB J. 31, 1254–1259. doi: 10.1096/fj.201700028, PMID: 28360375

[ref119] Selkoe D. J. (2011). Alzheimer's disease. Cold Spring Harb. Perspect. Biol. 3:a004457. doi: 10.1101/cshperspect.a004457, PMID: 21576255 PMC3119915

[ref120] Shen K. Meyer T. (1999). Dynamic control of CaMKII translocation and localization in hippocampal neurons by NMDA receptor stimulation. Science 284, 162–167. doi: 10.1126/science.284.5411.162, PMID: 10102820

[ref121] Shen K. Teruel M. N. Connor J. H. Shenolikar S. Meyer T. (2000). Molecular memory by reversible translocation of calcium/calmodulin-dependent protein kinase II. Nat. Neurosci. 3, 881–886. doi: 10.1038/78783, PMID: 10966618

[ref122] Sheng M. Hoogenraad C. C. (2007). The postsynaptic architecture of excitatory synapses: a more quantitative view. Annu. Rev. Biochem. 76, 823–847. doi: 10.1146/annurev.biochem.76.060805.160029, PMID: 17243894

[ref123] Shields S. M. Ingebritsen T. S. Kelly P. T. (1985). Identification of protein phosphatase 1 in synaptic junctions: dephosphorylation of endogenous calmodulin-dependent kinase II and synapse-enriched phosphoproteins. J. Neurosci. 5, 3414–3422. doi: 10.1523/JNEUROSCI.05-12-03414.1985, PMID: 3001244 PMC6565242

[ref124] Shifman J. M. Choi M. H. Mihalas M. Mayo S. L. Kennedy M. B. (2006). Ca^2+^/Calmodulin-dependent protein kinase II (CaMKII) is activated by calmodulin with two bound calciums. Proc. Natl. Acad. Sci. U. S. A. 103, 13968–13973. doi: 10.1073/pnas.0606433103, PMID: 16966599 PMC1599897

[ref125] Silva A. J. Paylor R. Wehner J. M. Tonegawa S. (1992). Impaired spatial learning in α-calcium-calmodulin kinase II mutant mice. Science 257, 206–211. doi: 10.1126/science.1321493, PMID: 1321493

[ref126] Singh D. Bhalla U. S. (2018). Subunit exchange enhances information retention by CaMKII in dendritic spines. Elife 7:7. doi: 10.7554/eLife.41412, PMID: 30418153 PMC6286124

[ref127] Sjostrom P. J. Nelson S. B. (2002). Spike timing, calcium signals and synaptic plasticity. Curr. Opin. Neurobiol. 12, 305–314. doi: 10.1016/S0959-4388(02)00325-2, PMID: 12049938

[ref128] Sneddon M. W. Faeder J. R. Emonet T. (2011). Efficient modeling, simulation and coarse-graining of biological complexity with NFsim. Nat. Methods 8, 177–183. doi: 10.1038/nmeth.1546 21186362

[ref129] Strack S. Barban M. A. Wadzinski B. E. Colbran R. J. (1997). Differential inactivation of postsynaptic density-associated and soluble Ca2+/calmodulin-dependent protein kinase II by protein phosphatases 1 and 2A. J. Neurochem. 68, 2119–2128. doi: 10.1046/j.1471-4159.1997.68052119.x, PMID: 9109540

[ref130] Strogatz S. H. (2018). Nonlinear dynamics and Chaos: with applications to physics, biology, chemistry, and engineering. Second Edition, Boca Raton, FL: CRC Press. 45–97.

[ref131] Tapia J. J. Saglam A. S. Czech J. Kuczewski R. Bartol T. M. Sejnowski T. J.. (2019). MCell-R: a particle-resolution network-free spatial modeling framework. Methods Mol. Biol. 1945, 203–229. doi: 10.1007/978-1-4939-9102-0_9, PMID: 30945248 PMC6580425

[ref132] Thiel G. Czernik A. J. Gorelick F. Nairn A. C. Greengard P. (1988). Ca^2+^/calmodulin-dependent protein kinase II: identification of threonine-286 as the autophosphorylation site in theasubunit associated with the generation of Ca^2+^-independent activity. Proc. Natl. Acad. Sci. U. S. A. 85, 6337–6341. doi: 10.1073/pnas.85.17.6337, PMID: 2842767 PMC281965

[ref133] Thomas M. J. Moody T. D. Makhinson M. O'Dell T. J. (1996). Activity-dependent b-adrenergic modulation of low frequency stimulation induced LTP in the hippocampal CA1 region. Neuron 17, 475–482. doi: 10.1016/S0896-6273(00)80179-8, PMID: 8816710

[ref134] Tse J. K. Giannetti A. M. Bradshaw J. M. (2007). Thermodynamics of calmodulin trapping by Ca2+/calmodulin-dependent protein kinase II: subpicomolar Kd determined using competition titration calorimetry. Biochemistry 46, 4017–4027. doi: 10.1021/bi700013y, PMID: 17352496

[ref135] Tullis J. E. Larsen M. E. Rumian N. L. Freund R. K. Boxer E. E. Brown C. N.. (2023). LTP induction by structural rather than enzymatic functions of CaMKII. Nature 621, 146–153. doi: 10.1038/s41586-023-06465-y, PMID: 37648853 PMC10482691

[ref136] Valtschanoff J. G. Weinberg R. J. (2001). Laminar organization of the NMDA receptor complex within the postsynaptic density. J. Neurosci. 21, 1211–1217. doi: 10.1523/JNEUROSCI.21-04-01211.2001, PMID: 11160391 PMC6762240

[ref137] Vargas-Caballero M. Robinson H. P. (2004). Fast and slow voltage-dependent dynamics of magnesium block in the NMDA receptor: the asymmetric trapping block model. J. Neurosci. 24, 6171–6180. doi: 10.1523/JNEUROSCI.1380-04.2004, PMID: 15240809 PMC6729657

[ref138] Walikonis R. S. Oguni A. Khorosheva E. M. Jeng C.-J. Asuncion F. J. Kennedy M. B. (2001). Densin-180 forms a ternary complex with the α-subunit of CaMKII and α-actinin. J. Neurosci. 21, 423–433. doi: 10.1523/JNEUROSCI.21-02-00423.2001, PMID: 11160423 PMC6763799

[ref139] Walkup W. G. Mastro T. L. Schenker L. T. Vielmetter J. Hu R. Iancu A.. (2016). A model for regulation by SynGAP-alpha1 of binding of synaptic proteins to PDZ-domain 'Slots' in the postsynaptic density. Elife 5:e16813. doi: 10.7554/eLife.16813, PMID: 27623146 PMC5040590

[ref140] Watanabe T. Huang H. B. Horiuchi A. da Cruze Silva E. F. Hsieh-Wilson L. Allen P. B.. (2001). Protein phosphatase 1 regulation by inhibitors and targeting subunits. Proc. Natl. Acad. Sci. U. S. A. 98, 3080–3085. doi: 10.1073/pnas.051003898, PMID: 11248035 PMC30610

[ref141] Watterson D. M. Harrelson W. G. Jr. Keller P. M. Sharief F. Vanaman T. C. (1976). Structural similarities between the Ca^2+^-dependent regulatory proteins of 3′:5′-cyclic nucleotide phosphodiesterase and actomyosin ATPase. J. Biol. Chem. 251, 4501–4513. doi: 10.1016/S0021-9258(17)33231-3, PMID: 181375

[ref142] Walkup W. G. Washburn L. Sweredoski M. J. Carlisle H. J. Graham R. L. Hess S.. (2015). Phosphorylation of synaptic GTPase-activating protein (synGAP) by Ca^2+^/calmodulin-dependent protein kinase II (CaMKII) and cyclin-dependent kinase 5 (CDK5) alters the ratio of its GAP activity toward ras and rap GTPases. J. Biol. Chem. 290, 4908–4927. doi: 10.1074/jbc.M114.614420, PMID: 25533468 PMC4335230

[ref143] Zador A. Koch C. Brown T. H. (1990). Biophysical model of a Hebbian synapse. Proc. Natl. Acad. Sci. U. S. A. 87, 6718–6722. doi: 10.1073/pnas.87.17.6718, PMID: 2168555 PMC54608

[ref144] Zhabotinsky A. M. (2000). Bistability in the Ca(2+)/calmodulin-dependent protein kinase-phosphatase system. Biophys. J. 79, 2211–2221. doi: 10.1016/S0006-3495(00)76469-1, PMID: 11053103 PMC1301111

