# Supplementary material for: A spatial model of autophosphorylation of CaMKII predicts that the lifetime of phospho-CaMKII after induction of synaptic plasticity is greatly prolonged by CaM-trapping
Source: Front Synaptic Neurosci. 2025 Apr 4;17:1547948. doi: 10.3389/fnsyn.2025.1547948 (PMC12006173; doi:10.3389/fnsyn.2025.1547948)
Supplement: Supplementary file 1 [file Data_Sheet_1.PDF]

Supplementary Material

Link to supplementary CaMKII_multiburst_color.mov file and legend; and to .tar.gz file containing the four model files (.blend) used in this work: http://www.mcell.cnl.salk.edu/models/spatial-model-of-CaMKII-2024-1/

Legend for "CaMKII_multiburst_color.mov":

The movie shows the activation of CaMKII holoenzyme within the cytoplasm of a dendritic spine between t=0.9 sec and t=2.1 sec, illustrated in Fig 3 in response to the stimulus shown in Fig 5. The stimulus begins at t=1.0 sec. The model shown in the movie includes all the molecules, reaction pathways, and resulting dynamics described in the manuscript. However, for clarity the movie visualizes only the freely diffusing Ca2+ ions (gold spheres), diffusing molecules of CaMKII holoenzyme (twin torus and sphere glyphs, with color scheme given below), and a patch of 15 NMDARs in the postsynaptic membrane (pentagonal receptor glyphs, with color scheme given below).

Changes in the state of the CaMKII holoenzyme and NMDARs are indicated by changes in color:

NMDAR

unbound: dark green

single Glu bound: medium green

double bound closed: bright green double bound open, unblocked by

Mg: white double bound open, blocked by Mg: red

desensitized: dark gray

CaMKII

CaMKII is a dodecamer composed of two hexameric rings (Fig 1). The six subunits of each ring can be autophosphorylated when activated. Here we represent each ring of the holoenzyme by a 2 piece glyph composed of a torus (whose color indicates the CaM-bound state of the hexameric ring) with a sphere at the center (whose color indicates the phosphorylation status of the whole ring). The dodecameric holoenzyme is represented by two of these torus-with-sphere assemblies stacked back-to-back. The color scheme is:

Torus representing a single hexameric ring:

0 CaM4 bound:	red
1 CaM4 bound:	green
2 CaM4 bound:	blue
3 CaM4 bound:	cyan
4 CaM4 bound:	magenta
5 CaM4 bound:	yellow
6 CaM4 bound:	white

Sphere representing the phosphorylation status of a single hexameric ring: 0 subunits phosphorylated: black

1 subunits phosphorylated:	red
2 subunits phosphorylated:	green
3 subunits phosphorylated:	blue
4 subunits phosphorylated:	cyan
5 subunits phosphorylated:	magenta
6 subunits phosphorylated:	yellow

Supplementary Methods.

Numbers of protein molecules.

<u>CaMKII.</u> The number of subunits of CaMKII in the median-sized hippocampal spine (#37) was set as follows. The average weight of a rat brain is ~ 1.4 g. A common assumption is that protein makes up about 10% of that weight, or 140 mg. Since ~80% of the volume of the brain is water and 1g of water has a volume of 1 ml, we assume that the average volume of a rat brain is 1.4 mls. Then the average protein concentration in the brain is ~140 mg/1.4 ml = 100 mg/ml. We found that CaMKII is highly concentrated in the brain (1). In the hippocampus it is ~2% of the total protein (2). Thus, its concentration by weight is ~ 2 mg/ml, averaged over hippocampal tissue. Since it is found almost entirely in neurons, we assume that its average concentration in hippocampal neurons is ~ 4 mg/ml. The average concentration of the ~600,000 kDal holoenzyme in hippocampal neurons is:

4 mg/ml x 1 mmole/600,000 mg x 1000ml/liter = 0.00667 mM or 6.67 µM. The average concentration of individual CaMKII subunits is 12 x 6.67 = ~80 µM.

The volume of the cytosol of spine #37 is 0.016 μm^3 (0.016 fl). So, the number of holoenzymes in the spine cytosol is:

~ 1.6 x 10-17 liters x 6.67 x 10-6 (moles/liter) x 6.02 x 1023 (particles/mole) ≈ 64.

We set the number of CaMKII holoenzymes in the spine to 60; and the number of individual CaMKII subunits to 720 (~80 µM).

<u>Calmodulin.</u> The concentrations of CaM in soluble and particulate fractions of brain were measured by Kakiuchi et al., 1982 (3). Estimates of the total concentration ranged from ~25 to ~35 µM. We added 30 µM CaM to the spine and to the attached cylinder. The initial number of CaM molecules added to the spine was 290. Because CaM was allowed to diffuse freely between the spine and the attached cylinder, the total number of CaM molecules (bound and free) in the spine increased during the stimulus as CaM bound to CaMKII and free CaM stayed constant. See Figs. 7S and 8.

<u>Protein Phosphatase-1.</u> The absolute concentration of PP1 in hippocampal spines is not well measured experimentally. We first estimated an overall concentration in brain from data in References (4) and (5). We then took into account the findings of Shields et al. (6), as well as Allen et al. (7) and Ouimet et al. (8), all of whom show that PP1 is highly concentrated in synaptic spines compared to overall brain cytosol.

Ingebritsen et al. (4) measured the overall specific activity of PP1 in brain tissue as 0.58 nmoles Pi/min/mg protein. If 1 liter of brain volume contains 100 g protein, we get an approximate average concentration of PP1 activity in the brain overall = 58,000 nmoles Pi/min/liter.

Watanabe et al. (5) measure the k_cat of PP1 as:

~11.5 Pi released /s/PP1 molecule = 690 Pi/min/PP1 molecule = 690 nmol Pi/min/nmol PP1.

Thus, we can calculate an approximate concentration of PP1 catalytic units in the brain overall as:

58,000 nmoles Pi/min/liter x 1 nmole PP1/690 nmol Pi/min = 84 nmoles/liter = 0.084 µM.

Because PP1 is considerably more concentrated in spines than in brain overall, we used a concentration of 1.25 µM as a starting point. We also simulated the effects of 0.65 µM, 2.5 µM and 5 µM.

Table S1: Parameter table with sources of reaction rates for the models of Ca^{2+} and CaM binding to CaMKII.

Description	Parameter	Value	Refs	Parameter	Value	Refs
Ca^{2+} binding to CaM	k_on1C	4 μM^{-1} s^{-1}	original refs. are listed in Pepke et al., 2010*	k_off1C	40.24 s^{-1}	original refs. are listed in Pepke et al., 2010*
	k_on2C	10 μM^{-1} s^{-1}	“	k_off2C	9.3 s^{-1}	“
	k_on1N	100 μM^{-1} s^{-1}	“, see notes	k_off1N	2660 s^{-1}	“
	k_on2N	150 μM^{-1} s^{-1}	“	k_off2N	990 s^{-1}	“
CaM binding to unphosphorylated and phosphorylated CaMKII (non-trapping model)<br><br>and<br><br>CaM binding to unphosphorylated CaMKII (initial low affinity site in trapping model)	k_on_laCaM0	3.8×10^{-3} $\mu M^{-1} s^{-1}$	“	k_offCaM0	6.56 s^{-1}	"
	k_on_laCaM1C	59×10^{-3} μM^{-1} s^{-1}	“	k_offCaM1C	6.72 s^{-1}	"
	k_on_laCaM2C	0.92 μM^{-1} s^{-1}	“	k_offCaM2C	6.35 s^{-1}	“
	k_on_laCaM1C1N	0.33 μM^{-1} s^{-1}	“	k_offCaM1C1N	5.68 s^{-1}	“
	k_on_laCaM2C1N	5.2 μM^{-1} s^{-1}	“	k_offCaM2C1N	5.25 s^{-1}	"
	k_on_laCaM1N	22 $\times 10^{-3}$ μM^{-1} s^{-1}	“	k_offCaM1N	5.75 s^{-1}	"
	k_on_laCaM2N	0.1 μM^{-1} s^{-1}	“	k_offCaM2N	1.68 s^{-1}	“
	k_on_laCaM1C2N	1.9 μM^{-1} s^{-1}	"	k_offCaM1C2N	2.09 s^{-1}	“
	k_on_laCaM4	30 μM^{-1} s^{-1}	"	k_offCaM4	1.95 s^{-1}	"
CaM binding to high-affinity site on CaMKII (trapping model)	k_on_haCaM0	2.28×10^{2} $M^{-1}s^{-1}$	***	k_off_haCaM0	0.1 s^{-1}	***
	k_on_haCaM1C	1.73×10^{4} M^{-1} s^{-1}	***	k_off_haCaM1C	0.05 s^{-1}	***
	k_on_haCaM2C	1.69 μM^{-1} s^{-1}	***	k_off_haCaM2C	0.03 s^{-1}	***
	k_on_haCaM1C1N	0.656 μM^{-1} s^{-1}	***	k_off_haCaM1C1N	0.03 s^{-1}	***
	k_on_haCaM2C1N	3.57 μM^{-1} s^{-1}	***	k_off_haCaM2C1N	1x10 s^{-1}	***
	k_on_haCaM1N	7.22×10^{-5} μM^{-1} s^{-1}	***	k_off_haCaM1N	0.05 s^{-1}	***
	k_on_haCaM2N	0.674 μM^{-1} s^{-1}	***	k_off_haCaM2N	0.03 s^{-1}	***
	k_on_haCaM1C2N	3.41 μM^{-1} s^{-1}	***	k_off_haCaM1C2N	1×10^{-3} s^{-1}	***
	k_on_haCaM4	50.0 μM^{-1} s^{-1}	***	k_off_haCaM4	9×10^{-5} s^{-1}	***

Ca^{2+} binding to CaM-CaMKII (non-trapping model)	k_on_K1C	44 μM^{-1} s^{-1}	original refs. are listed in Pepke et al., 2010*	k_off_K1C	29.04 s^{-1}	original refs. are listed in Pepke et al., 2010*
	k_on_K2C	44 μM^{-1} s^{-1}	"	k_off_K2C	2.52 s^{-1}	"
	k_on_K1N	75 μM^{-1} s^{-1}	"	k_off_K1N	301.5 s^{-1}	"
	k_on_K2N	76 μM^{-1} s^{-1}	"	k_off_K2N	32.68 s^{-1}	"
Ca^{2+} binding to CaM-CaMKII (trapping model) (low affinity site)	k_on_la1C	44 μM^{-1} s^{-1}	"	k_off_1C	29 s^{-1}	"
	k_on_la2C	44 μM^{-1} s^{-1}	"	k_off_la2C	2.5 s^{-1}	"
	k_on_la1N	75 μM^{-1} s^{-1}	"	k_off_la1N	315 s^{-1}	"
	k_on_la2N	75 μM^{-1} s^{-1}	"	k_off_la2N	31.8 s^{-1}	"
Ca^{2+} binding to CaM-CaMKII (trapping model) (high affinity site)	k_on_ha1C	44 μM^{-1}s^{-1}	"	k_off_ha1C	2.9 s^{-1}	"
	k_on_ha2C	44 μM^{-1}s^{-1}	"	k_off_ha2C	0.25 s^{-1}	"
	k_on_ha1N	75 μM^{-1}s^{-1}	"	k_off_ha1N	31.5 s^{-1}	"
	k_on_ha2N	75 μM^{-1}s^{-1}	"	k_off_ha2N	3.18 s^{-1}	"
Coarse-grained autophosphorylation rates for CaMKII	k_pCaMpartial	0.1 s^{-1}	Olwin et al., 1984; Pepke et al., 2010**			
	k_pCaM4	0.96 s^{-1}	"			
CaMKII dephosphorylation by PP1 (Michaelis-Menten constants)	k_catPP1	11.5 s^{-1}	(Watanabe et al., 2001)			
	k_mPP1	11 μM	(Bradshaw et al., 2003)			
	k_onPP1	10 μM^{-1}s^{-1}	Assumed	k_offPP1	98.5 s^{-1}	Calculated from Km

The parameters for transmitter release from the presynaptic terminal are listed in (9) and the parameters for NMDA receptor state changes, Ca^{2+}-channels, Ca^{2+} buffers, and Ca^{2+} pumps are listed in (10).

Notes: *The rates from (11) were adjusted to reflect the necessity for detailed balance as described in Pepke et al., 2010. **coarse-grained average phosphorylation rates for CaM-CaMKII with fewer than 4 Ca^{2+} bound and with 4 Ca^{2+} bound were taken from (11) and (12). *** The k_on for CaM4 prior to trapping was taken from (13), and the prolonged k_off for CaM4 in the trapped state was taken from (14). The Remaining k_off rates were estimated from Fig. 3 of (13), and the corresponding k-on rates were calculated from Kd's calculated to satisfy detailed balance. For the detailed balance calculations in the trapped state, we assumed a 10x higher affinity (10x Kd) of Ca^{2+} for CaM when CaM is bound to the high-affinity site of CaMKII, than when it is bound to the low affinity site. We assumed the higher affinity because the folding of the EF hands around bound Ca^{2+} is stabilized by the binding of CaM to the high-affinity site on CaMKII (i.e. see Fig. 2).

We did not use the rapid k_on rate for Ca^{2+} binding to CaM1N and CaM2N calculated in Faas et al. (15) because this rate is not compatible with molecular dynamics measurements (16) or with the rate of removal of water from Ca^{2+} ion (17-19).

The k_catPP1 was taken from (5). The k_mPP1 was taken from (20). k_off PP1 was calculated from the equation: $k\_offPP1 = k\_onPP1 * k\_mPP1 - k\_catPP1$.
k_onPP1 was assumed to be 10 μM^{-1} s^{-1}.

Figure S1. Channel opening kinetics in response to the stimulus shown in Figure 5.

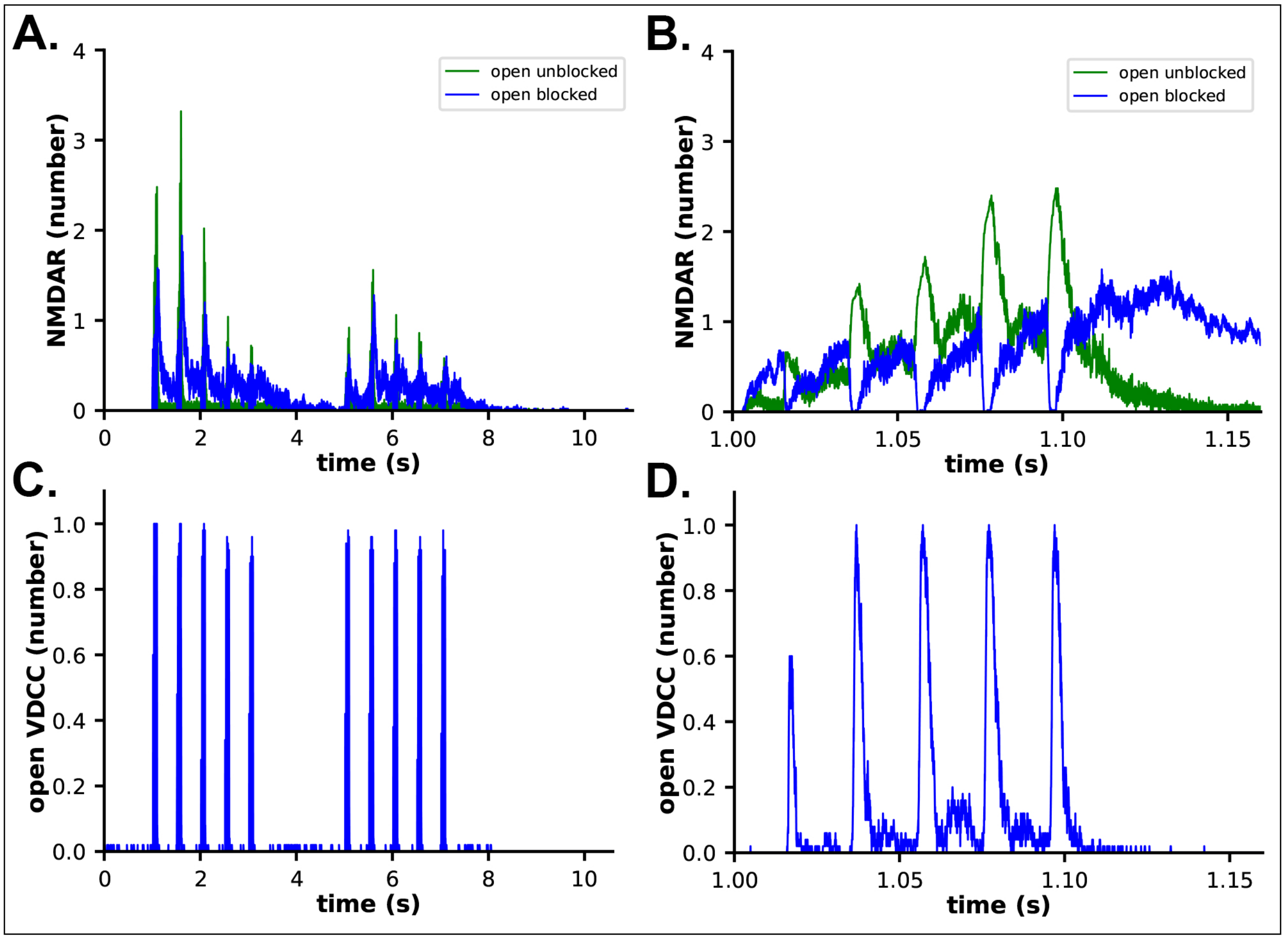

A). The number of open, unblocked NMDARs is shown in green, and open, blocked NMDARs in blue.

B). Data from A. between 1 and 1.15 s.

C). Kinetics of opening of the single VDCC on the spine membrane.

D). Data from C. between 1 and 1.15 s.

Data are averages of 50 simulations initiated with different random seeds.**Figure S3.** Ca^{2+}-bound states of free CaM during the two epoch stimulus.

Figure S2. Ca^{2+}-bound states of free CaM during the two epoch stimulus.

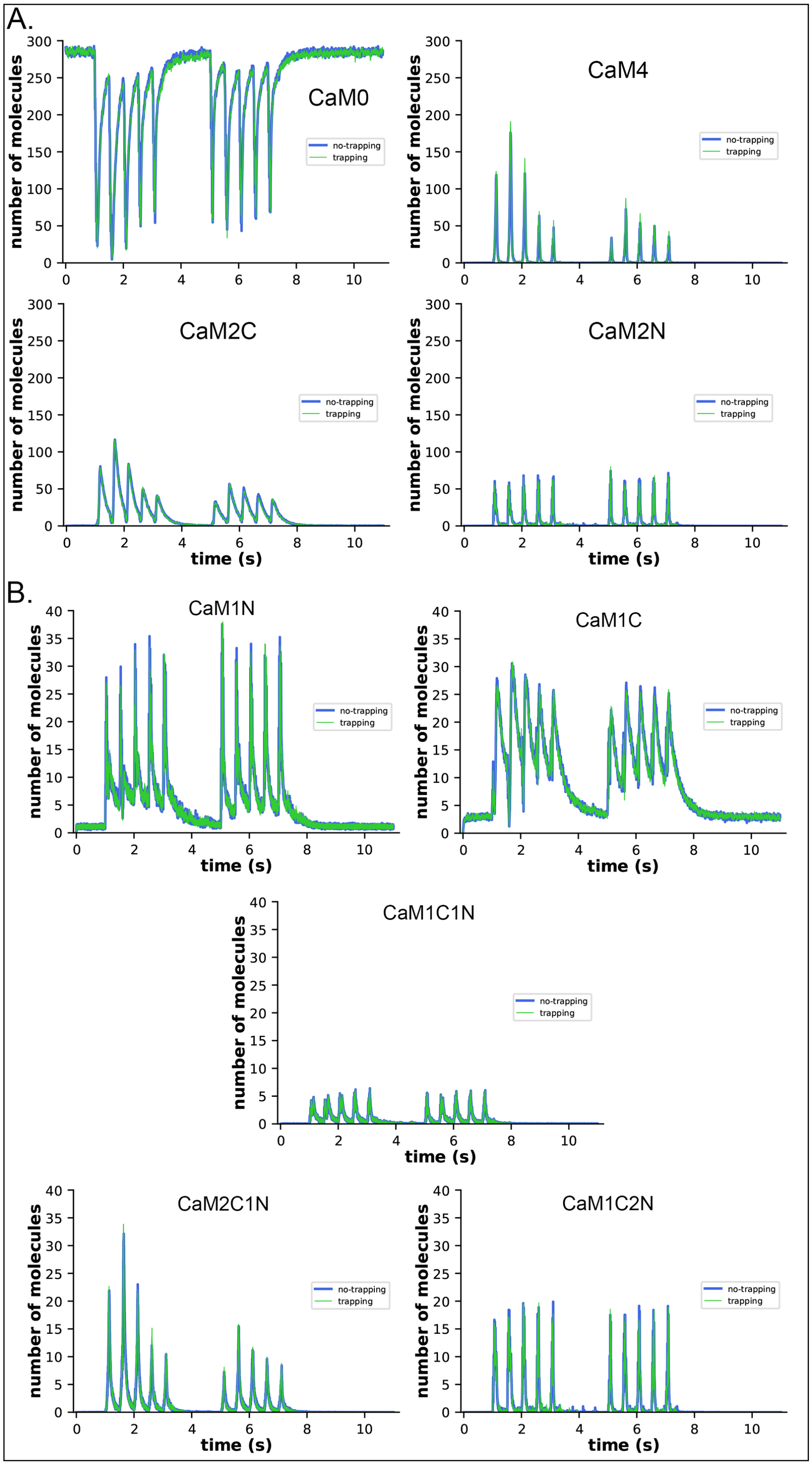

A). Free CaM0, CaM4, CaM2C and CaM2N plotted with ordinate of 300 molecules.
B). Free CaM1N, CaM1C, CaM1C1N, CaM2C1N, and CaM1C2N plotted with ordinate of 40 molecules.
Blue, no-trapping model; green, trapping model.

Figure S3. Binding of PP1 to pCaMKII subunits in simulations of the trapping model, in the absence of competition with CaM binding.

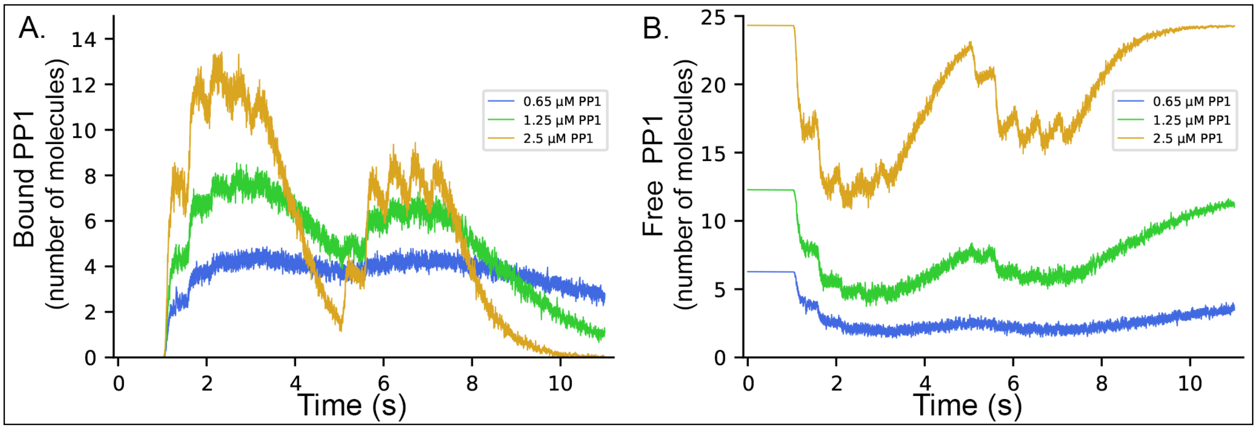

A). PP1 bound to CaMKII.
B). Free PP1. Blue, 0.65 µM PP1; Green, 1.25 µM PP1; Gold, 2.5 µM PP1.

Figure S4. Unphosphorylated CaMKII subunits (ucamkii) and CaM species bound to them during the two epoch stimulus.

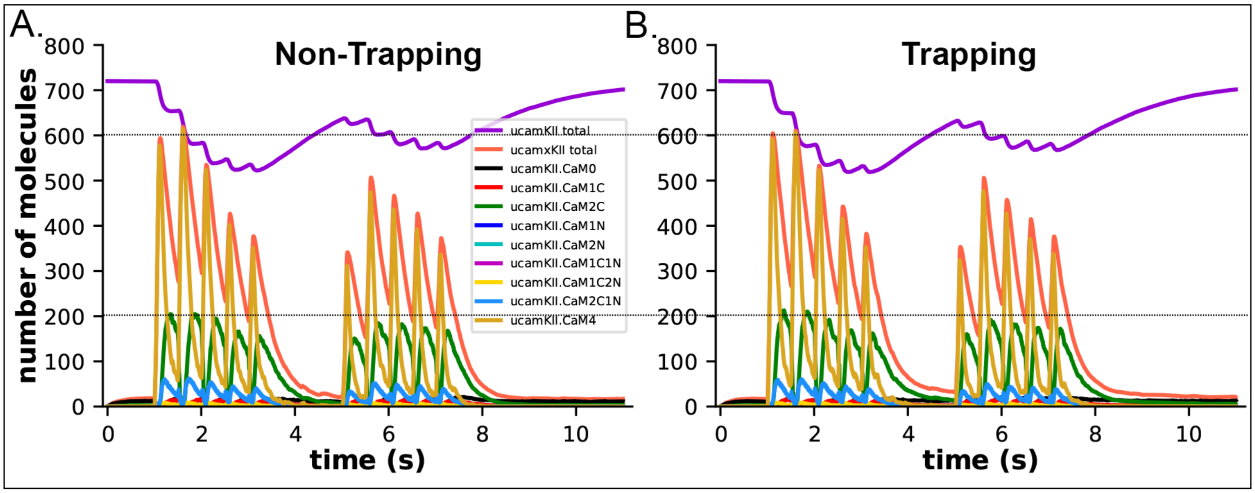

A). uCaMKII subunits and CaM species bound to them in simulations of the non-trapping model.
B). uCaMKII suubnits and CaM species bound to them in simulations of the trapping model.
Orange lines indicate the total CaM bound to unphosphorylated CaMKII subunits in each model. There is no significant difference between non-trapping and trapping models in the numbers of CaM species bound to uCaMKII subunits during the stimulus.

Bibliography

1. Bennett MK, Erondu NE, Kennedy MB. Purification and characterization of a calmodulin-dependent protein kinase that is highly concentrated in brain. J Biol Chem. 1983;258:12735-44.
2. Erondu NE, Kennedy MB. Regional distribution of type II Ca^{2+}/calmodulin-dependent protein kinase in rat brain. J Neurosci. 1985;5:3270-7.
3. Kakiuchi S, Yasuda S, Yamazaki R, Teshima Y, Kanda K, Kakiuchi R, et al. Quantitative determinations of calmodulin in the supernatant and particulate fractions of mammalian tissues. J Biochem. 1982;92(4):1041-8.
4. Ingebritsen TS, Stewart AA, Cohen P. The protein phosphatases involved in cellular regulation. 6. Measurement of type-1 and type-2 protein phosphatases in extracts of mammalian tissues; an assessment of their physiological roles. Eur J Biochem. 1983;132(2):297-307.
5. Watanabe T, Huang HB, Horiuchi A, da Cruze Silva EF, Hsieh-Wilson L, Allen PB, et al. Protein phosphatase 1 regulation by inhibitors and targeting subunits. Proc Natl Acad Sci U S A. 2001;98(6):3080-5.
6. Shields SM, Ingebritsen TS, Kelly PT. Identification of protein phosphatase-1 in synaptic junctions - dephosphorylaton of endogenous calmodulin-dependent kinase II and synapse-enriched phosphoproteins. J Neurosci. 1985;5:3414-22.
7. Allen PB, Ouimet CC, Greengard P. Spinophilin, a novel protein phosphatase1 binding protein localized to dendritic spines. Proc Natl Acad Sci U S A. 1997;94:9956-61.
8. Ouimet CC, Katona I, Allen P, Freund TF, Greengard P. Cellular and subcellular distribution of spinophilin, a PP1 regulatory protein that bundles F-actin in dendritic spines. J Comp Neurol. 2004;479(4):374-88.
9. Nadkarni S, Bartol TM, Sejnowski TJ, Levine H. Modelling vesicular release at hippocampal synapses. PLoS Comput Biol [Internet]. 2010 PMC2978677]; 6(11):[e1000983 p.]. Available from: http://www.ncbi.nlm.nih.gov/pubmed/21085682.
10. Bartol TM, Keller DX, Kinney JP, Bajaj CL, Harris KM, Sejnowski TJ, et al. Computational reconstitution of spine calcium transients from individual proteins. Front Synaptic Neurosci. 2015;7:article 17.
11. Pepke S, Kinzer-Ursem T, Mihalas S, Kennedy MB. A dynamic model of interactions of Ca^{2+}, calmodulin, and catalytic subunits of Ca^{2+}/calmodulin-dependent protein kinase II. PLoS Comput Biol. 2010;6(2):e1000675.
12. Olwin BB, Edelman AM, Krebs EG, Storm DR. Quantitation of energy coupling between Ca^{2+}, calmodulin, skeletal muscle myosin light chain kinase, and kinase substrates. J Biol Chem. 1984;259(17):10949-55.
13. Meyer T, Hanson PI, Stryer L, Schulman H. Calmodulin trapping by calcium-calmodulin dependent protein kinase. Science. 1992;256(5060):1199-202.
14. Putkey JA, Waxham MN. A peptide model for calmodulin trapping by calcium/calmodulin-dependent protein kinase II. J Biol Chem. 1996;271(47):29619-23.
15. Faas GC, Raghavachari S, Lisman JE, Mody I. Calmodulin as a direct detector of Ca2+ signals. Nat Neurosci. 2011;14(3):301-4.
16. Project E, Nachliel E, Gutman M. The dynamics of Ca2+ ions within the solvation shell of calbindin D9k. PLoS One. 2011;6(2):e14718.

17. Eigen M. Fast elementary steps in chemical reaction mechanisms. Pure Appl Chem. 1963;6:97-116.
18. Eigen M, Hammes GG. Elementary Steps in Enzyme Reactions (as studied by relaxation spectrometry). Adv enzymol relat areas Mol Biol. 1963;25:1-38.
19. Hille B. Ion channels of excitable membranes. 3rd ed: Sinauer; 2001.
20. Bradshaw JM, Kubota Y, Meyer T, Schulman H. An ultrasensitive Ca2+/calmodulin-dependent protein kinase II-protein phosphatase 1 switch facilitates specificity in postsynaptic calcium signaling. Proc Natl Acad Sci U S A. 2003;100(18):10512-7.
